# Investigating meteorological/groundwater droughts by copula to study anthropogenic impacts

**DOI:** 10.1038/s41598-022-11768-7

**Published:** 2022-05-18

**Authors:** Sina Sadeghfam, Rasa Mirahmadi, Rahman Khatibi, Rasoul Mirabbasi, Ata Allah Nadiri

**Affiliations:** 1 Department of Civil Engineering, Faculty of Engineering, University of Maragheh, P.O. Box 55136-553, Maragheh, East Azerbaijan Iran; 2 GTEV-ReX Limited, Swindon, UK; 3 Department of Water Engineering, Faculty of Agriculture, Shahrekord University, Shahrekord, Iran; 4 Department of Earth Sciences, Faculty of Natural Sciences, University of Tabriz, 29 Bahman Boulevard, Tabriz, East Azerbaijan Iran

**Keywords:** Climate sciences, Environmental sciences, Hydrology

## Abstract

A critical understanding of the water crisis of Lake Urmia is the driver in this paper for a basin-wide investigation of its Meteorological (Met) droughts and Groundwater (GW) droughts. The challenge is to formulate a data-driven modelling strategy capable of discerning anthropogenic impacts and resilience patterns through using 21-years of monthly data records. The strategy includes: (i) transforming recorded timeseries into Met/GW indices; (ii) extracting their drought duration and severity; and (iii) deriving return periods of the maximum drought event through the copula method. The novelty of our strategy emerges from deriving return periods for Met and GW droughts and discerning anthropogenic impacts on GW droughts. The results comprise return periods for Met/GW droughts and their basin-wide spatial distributions, which are delineated into four zones. The information content of the results is statistically significant; and our interpretations hint at the basin resilience is already undermined, as evidenced by (i) subsidence problems and (ii) altering aquifers' interconnectivity with watercourses. These underpin the need for a planning system yet to emerge for mitigating impacts and rectifying their undue damages. The results discern that aquifer depletions stem from mismanagement but not from Met droughts. Already, migration from the basin area is detectable.

## Introduction

The challenge for defining meteorological (Met) and groundwater (GW) droughts is topical research through the following approaches: (i) those based on drought indices, which refer to frequency analysis^1–3^; and (ii) those based on the balance equation^4^, which refers to the continuity equation alone. The first approach defines droughts as a deficit in water-related variables, such as precipitation or groundwater level (for wider definitions, see https://drought.unl.edu/Education/DroughtIn-depth/TypesofDrought.aspx), using Standard Precipitation Index (SPI)^5^ or Standard Groundwater Index (SGI)^6^ but these are not capable of studying the interactions between human activities and drought indices. The second approach is capable of studying such interactions (see^7^) by using the mass balance equation, but they include parameters that cannot be determined readily. These parameters (such as aquifer recharge and natural discharge) are inherently uncertain, and as such, balance equations are not capable of studying variations in drought within a basin without approximations. Thus, the second approach is avoided in this research investigation. Yet another technique is through trend analysis in timeseries, but their applications to the basin of Lake Urmia are not reviewed here as their defensibility depends on having long records of data, which is not the case here.

**Figure 1:**
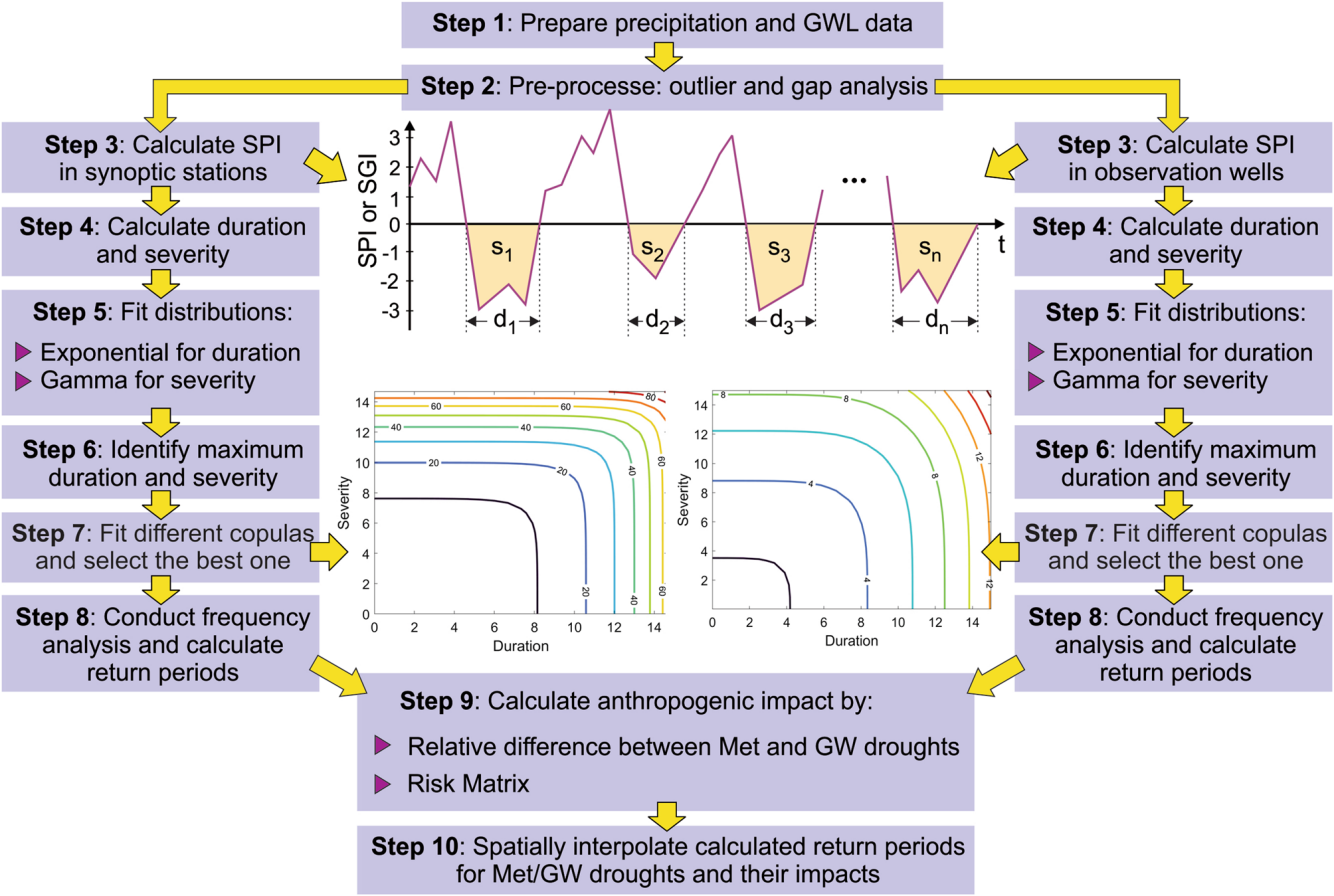
Modelling strategy and definition of drought duration (d) and severity (s).

The modelling strategy is pivotal for the novelty of the paper in terms of discerning the causes for aquifer depletion with possible causes comprising climate change, Met droughts, lack of an effective planning system and mismanagement. The key issues in the data-driven modelling strategy, depicted in Fig. 1, are now highlighted, in which the term maximum events is pivotal. As detailed in due course, the available sparse data comprise the precipitation records at 48 synoptic stations and groundwater levels at 158 observation wells, each with its 21 years of recorded timeseries at monthly intervals. Notably, a statistical summary of the synoptic stations and observation wells are given in the Electronic Information Data. Following Step 3 for each of Met/GW droughts, SPI/SGI timeseries are generated for each station/well, which serve to generate two more timeseries of duration and severity at each station/well (Step 5). Each of the generated timeseries has a maximum value, which is extracted to form one more basin-wide timeseries for Met droughts and GW droughts (Step 5), and as such, the study forms 48 values for duration/severity for Met droughts and 158 values for GW droughts. The application of Steps 5 and 6 to these data renders return periods, which are distributed over the study area using a spatial interpolation technique.

Met droughts are outcomes of natural processes but the role of carbon emission on Met droughts is global and not considered specifically here by the virtue of 21 years of data; whereas, GW droughts are impacted by both natural and anthropogenic factors. The impact of the Met drought on GW drought would take time, which should vary according to the capacity available in the aquifer but this is unknown for the aquifers in the study area. As the outcome of the modelling strategy is an emerging capability by putting Met droughts side-by-side of GW droughts, it becomes possible to ask the question that can interactions between Met/GW droughts be seen through data-driven modelling studies? To the best knowledge of the authors, there is no technique to investigate the questions, but the modelling strategy in Fig. 1 is a heuristic approach to discern anthropogenic impacts in terms of return periods. There is no precedence for the inter-comparison of Met/GW return periods, hence the learning from the results is ‘heuristic,’ as defined later.

Three techniques are used for the intercomparisons of the results: (i) visual comparisons; (ii) study relative differences between Met and GW return periods ((Met_T_-GW_T_)/GW_T_, where Met_T_ denotes the return period of Met drought and GW_T_ is that due to groundwater); and (iii) derive a ‘risk matrix’ for each aquifer depending on the mean of the return periods of Met/GW droughts. On a similar note, Ashraf et al. (2021) use the term ‘anthropogenic drought” as the ratio of anthropogenic withdrawal to the total outgoing flux using the balance equation^4^. Notably, heuristics refers to a clearly defined ‘scheme of doing things’ by any credible approach (further discussed by^8^).

The return periods are derived from bivariate distributions, which represent the mean time interval between drought events with the highest duration and severity observed in the recorded periods, where the term bivariate refers to duration and severity. The actual techniques are reviewed in due course but instead attention is drawn to delicate issues on drought studies that can be easily overlooked. By definition, a lower return period means a higher probability of occurrence. For floods, by definition, higher (or large) values of return periods stem from lower probability of occurrences, and this is generally understood to refer to adverse conditions; whereas, in drought studies, adverse conditions are captured by low values of return periods (higher frequencies and in plain language frequent droughts are adverse). Notably, drought studies set the condition to the event of the maximum conditions, as explained above. Thus, severe droughts are frequent droughts with maximum durations and maximum severities. Although SPI/SGI are derived individually at the synoptic stations and observation wells, the modelling strategy renders spatially-distributed return periods, which can be compared throughout the study area, and this serves as a heuristic basis for their inter-comparison. Comparing two stations with identical return periods does not mean identical drought events but it is the statement of the obvious that (i) Met droughts are unlikely to be impacted by GW droughts in the planning periods; (ii) GW droughts are likely to be impacted by mismanagement and by Met droughts.

Currently, Lake Urmia undergoes an unprecedented crisis as its water level has declined severely, most of which took place in the living memory during 2009–2016. The literature review on Lake Urmia is presented in the next section but its high-level summary is that it is an outcome of multiple factors including the absence a planning control system and mismanagement, lack of risk-based decision-making, anthropogenic and natural processes. The plethora of research outputs on the decline of lake water level (see the next section) is often focussed on climate change and/or drought but without a due regards to the absence of a planning system and mismanagement. Deriving return periods for Met and GW droughts provides the basis for a novel contribution by the paper, which uses the basin of Lake Urmia as the pilot study for the discernment of anthropogenic impacts on GW droughts. The discernment is facilitated by the modelling strategy in Fig. 1 and the emerging capability is novel and yet to become topical.

## Discussion of the findings and their implications

The results are presented in the next section but their salient findings are discussed below by interpreting them with respect to an identified zoning in droughts; identifying the baseline in the 1990s; evaluating the resilience of the basin under the current situation; relating the findings of the paper to other studies touching on policy issues in Iran and relating the thinking in the paper to the United Nations lead on sustainable development goals.

The heuristic zoning study to learn from return periods of Met/GW droughts, see the next section for results, by three approaches is based on the basic assumption that *if the return period of the Met drought at a location is equal to that of GW drought, the location is likely to be exposed to natural agents of drought, hence groundwater shortages ought to stem from Met droughts*. Such a location is deemed sustainable. However, if GW droughts are more frequent (of lower return period), this must be due to such factors as excessive water abstractions from aquifers and/or land use changes. Notably, land use is not under investigation in the paper, but Barideh and Nasimi^9^ demonstrate that agricultural lands in the basin of Lake Urmia have increased by 48% from 1987 to 2013. They also provide evidence that this increase is 13% in the period of the present study (2000–2021), which impacts the water demand abstracted from groundwater and consequently intensifies GW droughts. The results presented in the next section serve as evidence to explore the bearing of Met droughts on GW droughts in explicit terms through identifying the following zones (see Fig. 5):

(i) *Sustainable Zones* the results show no zone in the basin to have the return period of GW droughts to be significantly equal to its Met drought. Therefore, the aquifers in the basin are all impacted but under varying degrees, as explored below.
(ii) *Northern Aquifers* these aquifers are seen to be under relatively severe Met and GW droughts, although their relative differences fails to expose the severity of the problem. It is a challenge to discern the bearing of Met droughts and mismanagement on GW droughts in explicit terms but the challenge does not preclude the impacts of mismanagement.
(iii) *Central Aquifers (eastern and western aquifers)*: these are found under relatively severe Met and GW droughts although their values of return period are somewhat closer to each other. The degree of severity suggested by relative differences in return periods of Met/GW droughts and by their risk matrices are somewhat divergent, in which case it is necessary to resort to the principle of precaution. Nonetheless, these aquifers are deemed to be reaching the biting point toward aquifer depletion, where the scope for mismanagement is wide.
(iv) *Southern Aquifers (covering three major waterways of the basin):* the situation in this zone corresponds to relatively moderate Met drought regimes but relatively severe GW drought regimes, which confirm the aquifers to be under relatively severe anthropogenic stresses by interrupting the natural regime of water exchanges between the aquifers and watercourses reaching irreparable damages.

The results section discusses the correlation between Met/GW return periods and states that their overall correlation is poor and as such, Met droughts are not dominant factors on GW droughts. The authors are devising a more appropriate analytics to study the correlation within each zone by extending arithmetic calculus as developed by^11^.

The baseline for the environmental damage and impacts on Lake Urmia and its basin is relevant to understanding the results and building the overall mitigation plan. The problems were triggered circa 1990 at the onset of the green revolution in Iran, a period which coincided with the followings unprecedented changes: (i) population in the country rose from 39 million in 1980 to 56 million in 1990 and nearly 80 million in 2016; (ii) uncontrolled developments broke the backbone of traditional agricultural practices and opened the gateways to aquifer water abstraction practices by pumping and deep-well pumping; and (iii) the use of fertilisers have subsequently been contaminating the aquifers, although this is not the subject of investigation in the paper. The outcome was the ‘tragedy of the commons, which stemmed from a lack of long-term thinking, and this created an opportunity for individuals, rational or irrational, to reap the maximum benefit from their shared resource of aquifers. Soon the normal well pumps were not enough but the practice changed to using deep-wells.

Resilience in the basin is undermined as outlined here by interpreting the results in the light of the authors' professional knowledge of the local area. Three cases may be related to droughts impacting resilience, as follows. Depleting aquifers give rise to land subsidence, and its varying degrees have already been reported in relation to some of the aquifers surrounding the lake, see^12–14^. Subsidence undermines inherent land resilience and thereby undermine soil structures, agriculture, infrastructure, transport systems and buildings to the extent that future recoveries can be difficult if not impossible. Droughts, in general, have impacts on aquifer water quality through anthropogenic and geogenic processes, by reducing both aquifer storage and water quality; as well as causing over-abstraction^15^. Over-abstraction of groundwater is likely to alter (i) the dilution processes between stored water freshwater by recharging and (ii) the geogenic processes of maintaining rock-water ion exchanges, both of which have been observed in the aquifers of the basin^16–20^. As discussed in the next section, the past interactivities among the various hydrological components of the basin have largely been impacted in the sense that watercourses feed the aquifers when water is available but are not fed by aquifers anymore and these amplify environmental damage on the basin. Migration from the basin, with the recent memory of its lush valleys, is very concerning.

When the water crisis in Lake Urmia captured international attention, the urge for ‘saving face’ was turned into blaming climate change, where during 1995–2006 the global average temperature in the basin was approx. 0.5–1.0 °C rise (see^21^) but not enough to blame climate change. Even now climate change refers to the likely future possibilities but not to the present situation other than detecting some of its early signals. Currently, the average annual temperature rise in the basin of Lake Urmia is 1.5–1.6 °C (see^21^) but this is not still large enough to substantiate the onset of destructive forces of climate change. The blame culture prolongs the tragedy of the commons. In recent years, organisational arrangements have been put in place to cater for abstraction controls but these are operating nominally and shifting the blame game to the users without mitigating the problems by overhauling water use practices; as well as not producing publicly-communicated water resources plans, drought plans and water cycle management plans.

Drought studies are also reported by Maghrebi et al. (2021) concerning impacts on agriculture in Iran. They investigate the footprint of unsustainable development and report that during 1981–2013 increased agricultural activities gave rise to increased productivity but reduced water availability^22^. However, in time, depleting groundwater and surface water would instigate the process for reduced water/food security, and undermine environmental resilience. They also conclude that urgent policy reforms are required to create a balance between agricultural activities and water availability, else current policies expose the people to the risk of insecurity, job losses, migration, conflicts and tension.

The UN Sustainable Development Goals (SDGs) are one way of avoiding the tragedy of the commons, which are outcomes of scientific learning from past failures towards a more sustainable future. Delivering SDGs are now a common purpose in the global agenda and comprises 17 SDGs, broken down to some 169 targets (each of them are described in UN sites, see: https://unstats.un.org/sdgs/metadata/ or https://www.concernusa.org/story/sustainable-development-goals-explained/). The goals and targets are transformed into several hundred indicators which are being used to measure progress towards achieving the goals and targets^8^. The issue of droughts is mentioned in Targets 2.4 and 15.3; Paragraph 14 of *Our Shared Principles*; Paragraph 33 of the *New Agenda*; and Paragraphs 205, 207 and 208 of *the Future We Want*. The study underpins the need for sustainable development in the basin of Lake Urmia to stop the ongoing damages before they become irreparable.

The paper shows that return periods on their own are capable of giving a heuristic insight into drought problems. These may be seen as the evidence for the proof of concept for the modelling strategy to discern the role of anthropogenic impacts on aquifer droughts. The authors are now expanding their works in a number of directions including: (i) investigating a synthetic index to better summarise the problem; (ii) assessing impacts of climate change both on precipitation and on the aquifers of the Lake Urmia basin and thereby on Met/GW droughts; and (iii) introducing indices to gain an insight into aquifer droughts towards resilient and sustainable performances.

## Results

### An insight into the catastrophe of Lake Urmia

To date, the surface area of Lake Urmia is 2558 km^2^, which is shrunk to 51% of its value in 2000 and the measures by the government to restore the lake is still uncertain. A saltpan of approximately 1 m deep is left behind the shrunken lake, the health impacts of which are evident but not yet documented. However, the depleting aquifers are already showing land subsidence problems at some of these aquifers, see^12–14^. Khatibi et al.^23^ take an overview of the past initiatives on restoring Lake Urmia and note that any material solution is yet to emerge from all those apparent project proposals.

Unsustainable groundwater abstractions threaten water availability in Iran, which is evidenced by doubling abstraction wells in 13 years (2002–2015) (see^24^). Ashraf et al. (2021) demonstrate that the basin-scale groundwater droughts in Iran are affected by groundwater withdrawal by humans even though Met droughts intensify GW droughts^4^. Of the plethora of studies on Lake Urmia, Khatibi et al. (2020) study the decline of water levels at Lake Urmia and conclude that it is the outcome of mismanagement^23^; Vahedoost and Aksoy (2021) study the water balance in Lake Urmia in conjunction with the surrounding aquifers and surface water inflows and conclude that groundwater and evaporation are significant variables on the decline in the water level of Lake Urmia but the effect of groundwater surpasses all other variables^25^. Attributing the decline to droughts is not rare but there is no reason to assume that there is any prolonged extreme drought in the region other than operational water shortages during peak demands. Indeed, there has been exceptionally high precipitations e.g. in 2006–07 and 2018–19, significantly raising the water levels in the lake, when floodwaters were allowed to bypass the vast number of dams.

The hydrological cycle of the Lake Urmia basin has been encroached since the 1990s both in terms of surface water hydrology and groundwater exploitations^23^. There is no published study of the baseline conditions on drought occurrences in the study area with only some sporadic record of precipitation and GW levels. However, Khatibi et al.^23^ and Khatibi and Nadiri (2020) ^26^ hint at the pristine natural conditions prior to the 1990s, when traditional agricultural activities were woven over the natural resources in a sustainable fashion. Since the 1990s, mismanagements of resources have not been controlled and this remains the norm to date. The results produced by the paper and presented below examine critically the wisdom of attributing the crisis of Lake Urmia to meteorological droughts or climate change.

The scope of the dependence of water level in Lake Urmia on direct precipitation, streamflows, groundwater flows and baseflow (compensation flows or their absence) has received some attention, see^27^, according to which the Lake level may only depend by as much as 10% on direct precipitation; whereas water resources over-abstraction and the construction of dams are major contributors to the water level decline of the lake. Alizade Govarchin Ghale et al. (2018) compared the water balance change of Lake Urmia and SPI and observed that anthropogenic activities are 80% more effective on the lake water decline than the climatic changes accounting for as little as 20%^28^. They list the anthropogenic activities, which include over-abstraction of groundwater and surface water resources, developing agricultural lands and mismanagement of water resources to a severe encroachment onto the natural regime of the basin, both onto the lake water levels and groundwater levels. Subsequent to the risk realisations in 2008–2015 of the encroachments, there were no re-examination of the past decisions but more ambitions plans were proposed, which include the following: (i) water transfer from the Caspian Sea were discussed to restore the water levels in the lake but no steps were taken to this end; (ii) pilot schemes were nominated in 2014 for the artificial recharging of the aquifers by the West Azerbaijan Water Authority entitled “Groundwater Rehabilitation and Balancing;” and (iii) in the East Azerbaijan province pilot schemes were initiated to artificially recharge aquifers (four basins in the Shabestar aquifer from 1996 and one basin in the Azershahr aquifer from 2003).

The aim of the “Groundwater Rehabilitation and Balancing initiative was to stop the total over-abstractions and then, within 20 years, compensate cumulatively by reducing deficits in the aquifers and by bringing them to their baseline conditions through 15 projects (the Ministry of Agriculture: 3 projects; the Geological Survey Organization: 1 project; and the Ministry of Energy: 11 projects). Among the main projects being implemented by the Ministry of Energy aimed to address some issues [https://www.wsanw.ir/cs/Articles/18/291] including: (i) establish and strengthen water resources patrol teams; (ii) control, monitor and exploit unauthorised wells that are harmful to public interests; (iii) install volumetric and smart gauges and create a system for monitoring and controlling groundwater abstraction; (iv) prepare online water balance sheet and database of water resources; (v) organise authorised drilling companies and install GPS devices on drilling rigs; (vi) modify licenses for authorized agricultural wells based on the national water document and apply adjustment coefficient in order to achieve programmable groundwater.

To the best of the authors’ knowledge, the projects went to the backburner apparently due to the lack of financial resources and no strong support to the project; as well as the absence of bottom-up management strategies and participatory decision-making did not trigger any serious challenges. Overall, these plans have not been effective enough and some of the reasons include: lack of stable financial support for repairing and dredging, lack of macro-planning and goal setting both at the level of scientific centres and at the level of executive bodies, lack of a framework for the participation of farmers and rural councils for efficient water usage, prevention of soil erosion and the maintenance of watercourses and canals. The outcome is that the aquifers are being depleted and there is no drought plans, as overviewed below.

Drought planning in developed countries are through publicly communicated plans put in place for the delivery of various goals. For instance, in the UK, the aim is to safeguard the environment during drought and to oversee actions to secure public water supplies over often one year period. There are also severe and extreme droughts, as they become more prolonged that can be considered as part of drought planning. There are no known plans of this nature for managing Lake Urmia and its basin. An understanding of the occurrences of droughts in the Lake Urmia basin is a challenge since Lake Urmia has undergone a severe water level decline during 2009–2016 due to extensive program of constructing embankments on its watercourses. It is not scientific to attribute the decline to droughts or climate change without focussing on the extensive number of dams constructed since the 1990s. Nonetheless, the results presented in the section leads to answering the question that: did the Met droughts induce GW droughts; or do anthropogenic activities intensify the usual water shortage problem; and under what mechanisms do these activities intensify the decline in Lake Urmia and its basin? Without clear responses to these question, resilience planning is unlikely to be effective.

### Data, processed datasets and information contents of the results

Precipitation and groundwater level timeseries with 21 years length (2000–2020) are incorporated in this study for analysing meteorological and groundwater droughts, respectively. Although Tong et al. (2015) discusses the uncertainty within copula’s results increases when the data availability is reduced from 100 year-data to 20 year-data^29^, arguably still there are significant information signals within 21 years of data availability. For some stations in the study, the data availability is 30 or more, but 21 years of data are available for all of the stations and therefore all the results are produced for the common time base of 21 years. These are quite sparse but are all that is available. Data examinations in the pre-processing stage indicated that there were no outliers based on using the interquartile range^30^; also, the gaps in the data for all synoptic stations were less than 5% of the total data, estimated by fitting regression equations between a station with data gaps and other stations, rendering a correlation coefficient greater than 0.9. This justified the gap-filling algorithm; hence the pre-processed timeseries are deemed fit-for-purpose in calculating SPI and SGI.

Both SPI and SGI are calculated using the gamma distribution as per recommendations in the literature^6,31–33^. For SPI, the use of the gamma distribution seems widespread but for SGI there are recommendations to select a more appropriate distribution for a particular dataset. In the present study, the objective was not to identify the most accurate distribution but a fit-for-purpose distribution is deemed sufficient and this assumption was tested by the Kolmogorov–Smirnov test. The modelling strategy shown in Fig. 1 produces the results summarised in Table 1.

**Table 1:** Meteorological drought: a summary of statistical parameters for the fitted marginal distributions and copulas in 48 synoptic stations.

Station	Copula	*λ*	*α*	*β*	*θ*_*opt*_	Log-likelihood	NSE	RMSE	Station	Copula	*λ*	*α*	*β*	*θ*_*opt*_	Log-likelihood	NSE	RMSE
1	Frank	3.5	1.05	2.59	7.90	− 139.1	0.72	0.14	25	Clayton	3.5	1.43	1.48	1.06	− 107.8	0.89	0.09
2	Frank	3.0	0.77	4.43	8.22	− 124.2	0.72	0.14	26	Clayton	3.5	1.29	1.43	0.98	− 99.1	0.88	0.09
3	Galambos	3.5	0.88	2.73	2.72	− 101.5	0.73	0.14	27	Galambos	4.0	1.39	1.65	1.91	− 100.8	0.89	0.09
4	Frank	3.0	0.99	1.74	8.59	− 112.3	0.66	0.15	28	Clayton	3.5	2.38	0.96	0.46	− 105.6	0.77	0.12
5	Frank	3.5	0.71	4.07	7.45	− 133.9	0.67	0.15	29	Clayton	3.0	2.72	0.68	0.42	− 92.6	0.58	0.16
6	Frank	3.0	1.41	1.55	8.24	− 118.1	0.65	0.16	30	Clayton	3.5	0.93	2.62	0.98	− 111.6	0.66	0.15
7	Frank	3.0	0.95	3.64	11.67	− 105.7	0.55	0.18	31	Clayton	3.5	1.29	1.60	0.99	− 109.2	0.82	0.11
8	Frank	3.0	0.77	2.78	7.62	− 114.6	0.51	0.18	32	Clayton	3.5	1.01	2.15	0.69	− 108.9	0.78	0.12
9	Galambos	3.0	1.78	1.03	2.29	− 81.7	0.90	0.09	33	Galambos	2.5	0.88	2.68	2.02	− 112.7	0.87	0.09
10	Galambos	3.0	0.96	1.95	2.39	− 92.9	0.88	0.10	34	Frank	2.5	0.96	2.63	7.21	− 133.4	0.72	0.14
11	Clayton	2.5	1.17	1.25	0.65	− 98.4	0.87	0.11	35	Frank	3.0	1.34	1.40	8.30	− 114.3	0.75	0.14
12	Frank	2.5	1.02	1.97	7.28	− 120.5	0.60	0.18	36	Frank	2.5	1.03	2.11	5.72	− 131.0	0.65	0.16
13	Frank	2.5	1.38	1.65	6.38	− 99.9	0.65	0.16	37	Galambos	3.5	0.84	2.79	1.47	− 111.9	0.78	0.13
14	Frank	3.0	0.98	2.47	7.30	− 132.3	0.51	0.19	38	Frank	2.5	1.40	1.73	5.99	− 136.9	0.59	0.18
15	Galambos	3.5	0.98	2.38	2.58	− 92.1	0.86	0.10	39	Plackett	3.0	1.07	1.87	20.00	− 89.0	0.90	0.09
16	Galambos	3.0	1.20	1.76	2.11	− 113.2	0.87	0.10	40	Plackett	3.0	1.61	1.16	20.00	− 86.1	0.95	0.06
17	Frank	3.0	0.94	2.68	13.17	− 109.7	0.92	0.08	41	Galambos	3.5	0.95	1.90	2.68	− 82.6	0.90	0.08
18	Galambos	2.5	1.76	1.01	2.20	− 88.7	0.93	0.07	42	Clayton	3.5	0.96	2.63	1.15	− 113.7	0.84	0.10
19	Galambos	2.5	0.79	2.31	3.02	− 87.3	0.94	0.07	43	Galambos	3.5	0.67	5.15	2.96	− 102.5	0.88	0.09
20	Frank	3.0	1.05	2.10	9.81	− 117.7	0.90	0.08	44	Galambos	3.5	0.93	3.46	2.95	− 103.5	0.93	0.07
21	Frank	3.0	1.14	1.76	12.94	− 84.2	0.84	0.10	45	Clayton	3.5	1.21	2.10	1.11	− 112.3	0.87	0.10
22	Frank	2.5	1.09	2.02	9.61	− 120.8	0.84	0.10	46	Clayton	3.5	1.11	1.88	1.06	− 114.1	0.85	0.10
23	Galambos	4.0	1.00	2.43	2.33	− 118.6	0.72	0.15	47	Clayton	3.0	1.62	1.18	0.76	− 98.3	0.88	0.09
24	Frank	3.0	1.00	2.16	8.59	− 112.6	0.70	0.15	48	Clayton	3.0	0.70	3.93	1.44	− 117.2	0.85	0.10

The results in Table 1 show the details of the fitted copulas to monthly precipitation data for 48 synoptic stations; the details of which comprise: (i) select the best copula among its seven types of Clayton, Ali-Mikhail-Haq, Farlie-Gumbel-Morgenstern, Frank, Galambos, Gumbel-Hougaard, and Plackett (see^34^); (ii) identify the parameters related to their inherent marginal distributions (*λ*, *α*, *β*) using the data related to duration and severity, in which the exponential distribution is used for duration and the gamma distribution for severity, as discussed in Methods; (iii) optimise copula dependency parameter (*θ*) based on the method of Inference Function for Margins (IFM); (iv) calculate the logarithmic likelihood values corresponding to the optimised *θ*; and (v) calculate Nash–Sutcliffe Efficiency (NSE) and Root Mean Square Errors (RMSE) values by comparing fitted and empirical copulas. NSE varies in the range of 0.51–0.95 and RMSE in the range of 0.06–0.19. These serve as some evidence that the fitted copulas are fit-for-purpose and information content of the modelled return periods are statistically significant.

Similar to Table 1, the fitted copulas for the data of the 158 observation wells are presented in Table 2 using groundwater levels. For brevity, all the results are reported in the form of mean and standard deviations for each aquifer. The table also represents the best copula types and the number of corresponding observation wells but the other details are defined as for Table 1. The preliminary investigations with respect to fitting different distributions to duration and severity show that the exponential distribution is more appropriate to duration and the gamma distribution for severity of GW droughts. The table shows the means of NSE per aquifer, which vary in the range of 0.59–0.85 and that of RMSE in the range of 0.09–0.17. These serve as some evidence that the fitted copulas are fit-for-purpose and information content of the modelled return periods are statistically significant.

**Table 2:** Groundwater drought: statistical parameters for the fitted marginal distributions and copulas in 158 observation wells.

Aquifers and number of observation wells	Number of observation wells related to each type of Copula						*λ*		*α*		*β*		*θ*_*opt*_		Log-likelihood		NSE		RMSE	
	Clayton	AM Haq	FGM	Frank	Galambos	Gumbel H	Mean	St dv	Mean	Stdv	Mean	Stdv	Mean	Stdv	Mean	Stdv	Mean	Stdv	Mean	Stdv
Tasuj (8)	3	0	0	1	4	0	15.4	9.3	0.7	0.3	27.9	33.0	6.0	3.9	− 38.7	22.8	0.71	0.17	0.13	0.04
Shabestar (8)	2	0	0	5	1	0	9.7	5.2	1.2	1.2	18.7	18.6	9.6	5.6	− 41.3	27.0	0.68	0.16	0.13	0.04
Tabriz (16)	1	0	0	8	7	0	6.5	3.1	1.2	1.0	10.6	8.4	7.1	5.1	− 64.1	27.2	0.75	0.13	0.12	0.04
Azershahr (7)	0	0	0	5	2	0	8.6	4.2	0.9	0.4	12.3	10.6	9.7	6.7	− 70.3	29.5	0.71	0.20	0.14	0.05
Shiramin (3)	0	0	0	2	1	0	13.3	7.2	0.8	0.4	24.1	25.6	11.1	7.8	− 63.9	24.3	0.65	0.25	0.15	0.05
Ajabshir (6)	0	0	0	3	3	0	12.0	6.1	0.8	0.4	23.4	30.4	8.2	6.5	− 59.2	18.9	0.59	0.25	0.17	0.06
Maragheh-Bonab (13)	2	0	0	4	7	0	8.7	5.5	0.9	0.6	12.3	7.7	6.9	5.8	− 53.4	27.0	0.78	0.14	0.11	0.03
Miandoab-(Qoshachay) (23)	1	1	0	12	8	1	8.2	5.7	1.3	1.0	10.9	14.5	6.9	5.6	− 56.3	21.2	0.75	0.15	0.12	0.04
Mahabad (10)	2	0	0	8	0	0	10.5	2.4	0.5	0.1	25.0	5.8	14.6	4.7	− 51.0	14.5	0.65	0.06	0.15	0.02
Naghadeh (Sulduz) (20)	1	0	0	6	13	0	5.1	1.3	1.2	0.6	7.2	3.6	4.3	5.0	− 74.3	10.1	0.85	0.10	0.09	0.03
Urmia (28)	1	0	0	8	19	0	6.4	3.7	1.2	0.7	7.7	4.8	5.6	5.8	− 64.7	20.6	0.84	0.11	0.10	0.03
Kahriz (4)	0	0	1	1	2	0	7.1	4.3	1.9	1.8	10.2	14.2	6.2	9.2	− 47.5	29.4	0.77	0.24	0.11	0.05
Salmas (12)	4	0	0	3	4	1	6.6	2.9	1.7	1.4	4.9	4.6	4.4	3.9	33.4	26.5	0.71	0.18	0.12	0.03

**Figure 2:**
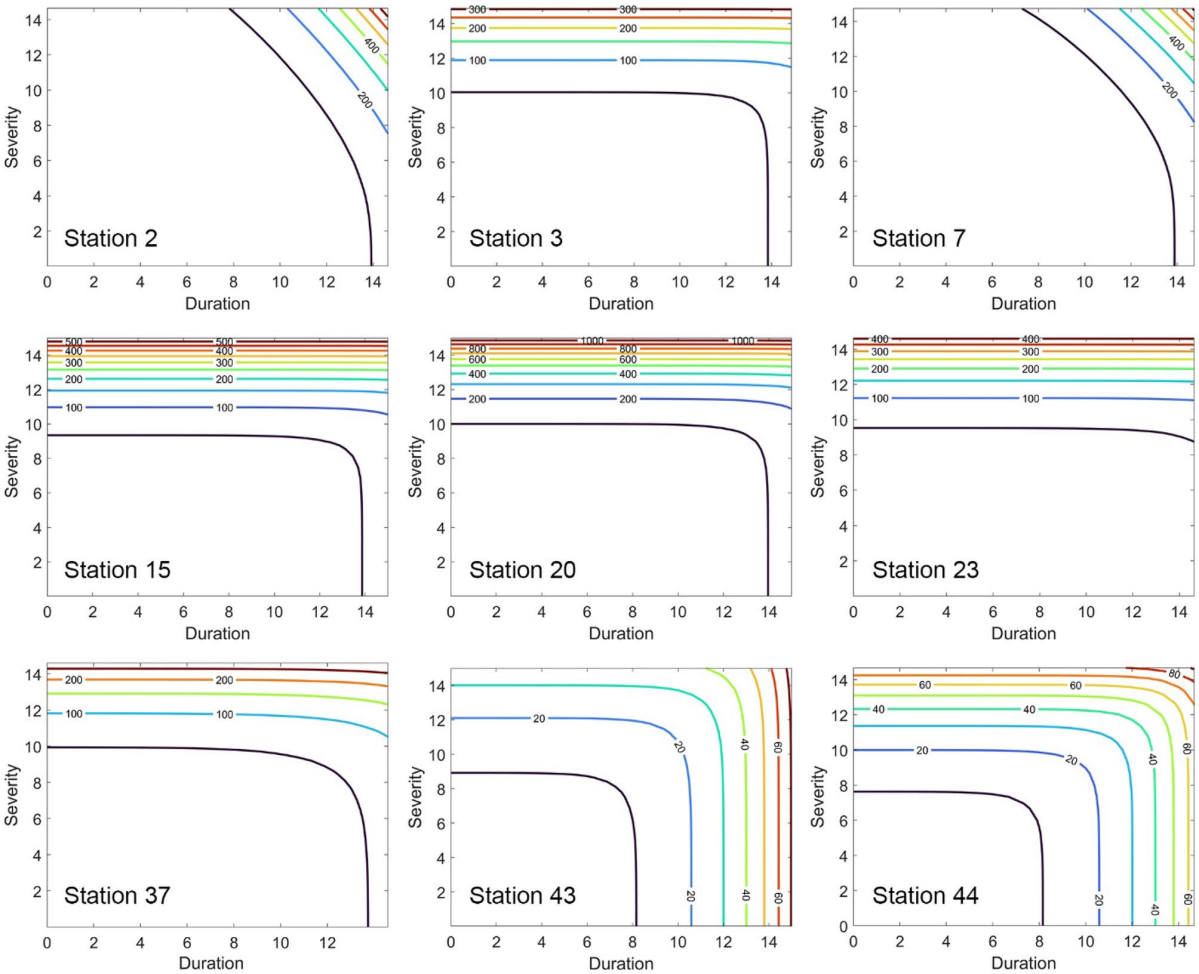
Bivariate return period for Met drought for a sample of stations based on fitted copulas.

**Figure 3:**
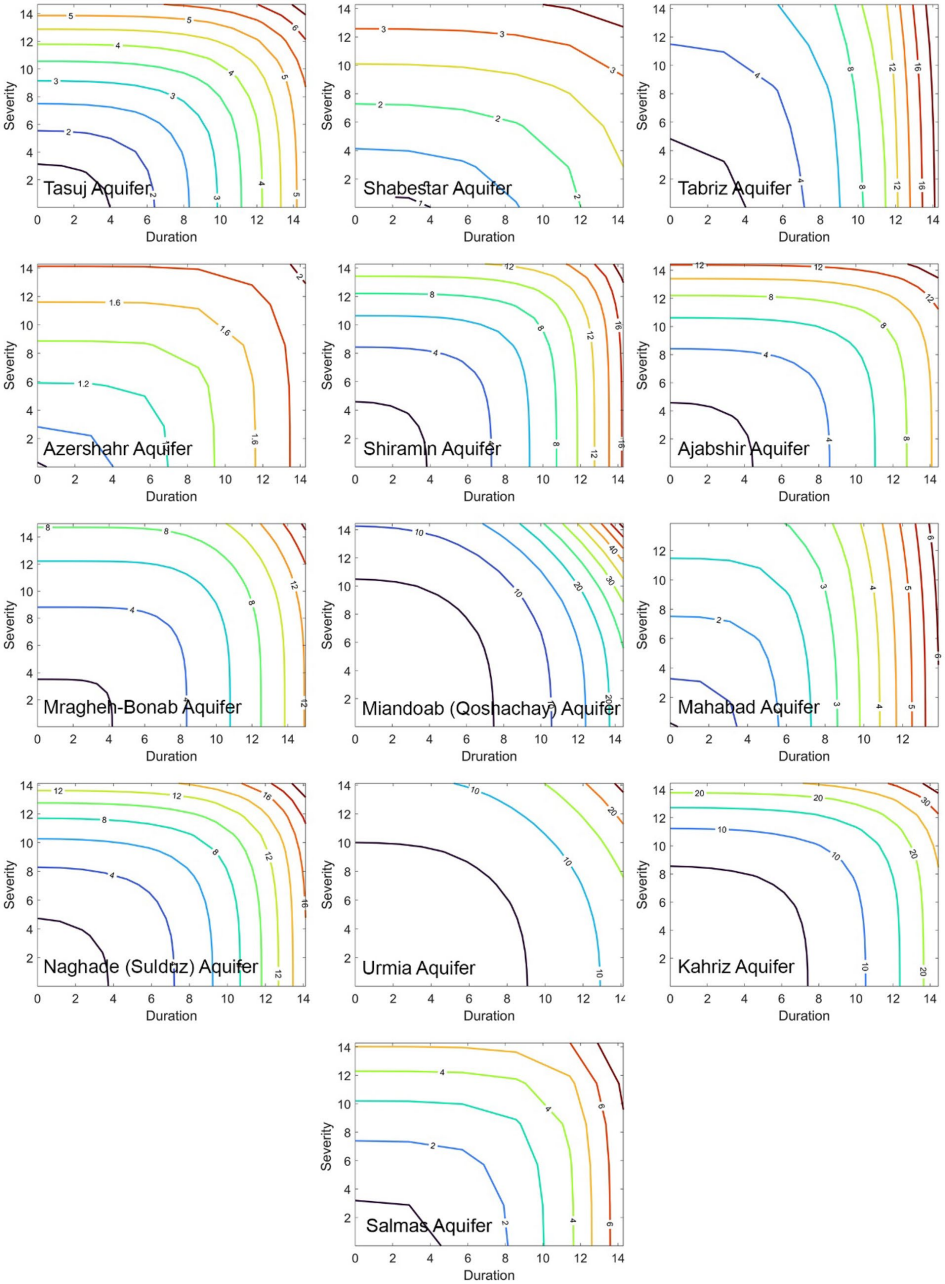
Bivariate return period for GW drought for a sample of observation wells based on fitted copulas.

**Figure 4:**
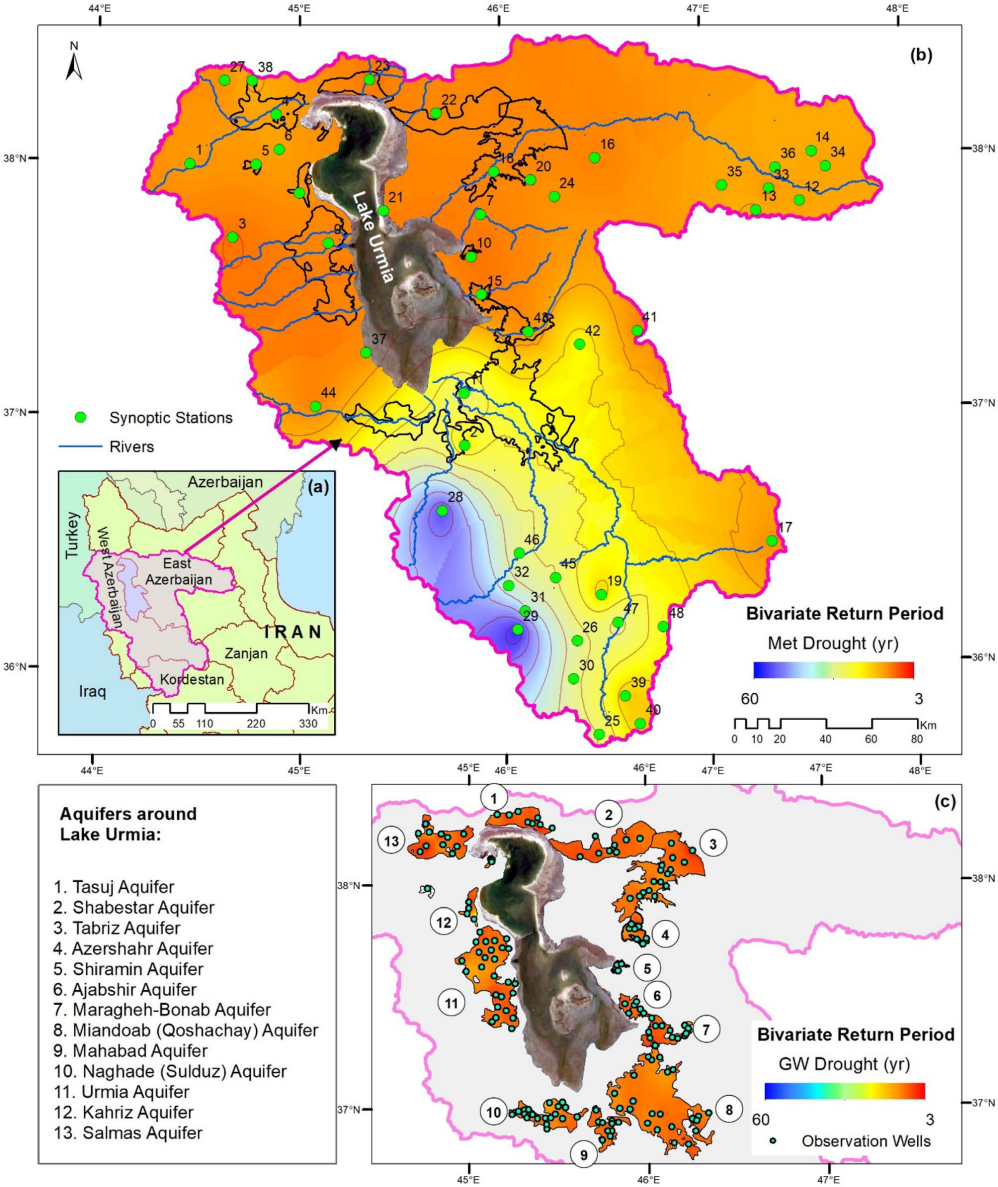
(**a**) Location map of the study area; (**b**) spatial distribution of Met drought return period within the Lake Urmia basin; and (**c**) spatial distribution of GW drought return period within the aquifers around the lake. Note 1: The contours for each aquifer varies but not much and more often are lower (more severe) than those of Met droughts. Note: 2: The figure is produced by the authors using QGIS 3.01 v 2018.

The copulas in Tables 1 and 2 are employed to carry out frequency analysis and obtain the return period of drought events corresponding to the maximum duration and severity in the available historical data. The bivariate return periods using the fitted copulas in terms of duration and severity are displayed in Fig. 2 for Met drought at a sample of synoptic stations and a similar sample is displayed in Fig. 3 for GW drought. The spatial distributions of copula-based bivariate return periods are given in Fig. 4 in two sets: (i) return period values for Met droughts within the basin of the lake; (ii) and that for GW droughts within each of the 13 aquifers around the lake. The identical red-to-blue colour palette for both types of droughts corresponds to low-to-high return periods.

### Overview of the results

To understand the results better, the physical system is subdivided into: (i) the lake, (ii) the surrounding aquifers, (iii) streamflows due to quick runoff, (v) interflows due to percolations; (iv) baseflows originating lost by aquifers or gained by them; and (vi) possible connectivity between the lake and its surrounding aquifers. A further issue of the primary importance is the compensation flows that must be allowed from the dams to maintain the ecological functions at their downstream. The overwhelming perception is that the embankment dams retain all the water and very little compensation flow or none is allowed to flow through, unless there is a risk of over-spillage. These are discussed in due course.

**Figure 5:**
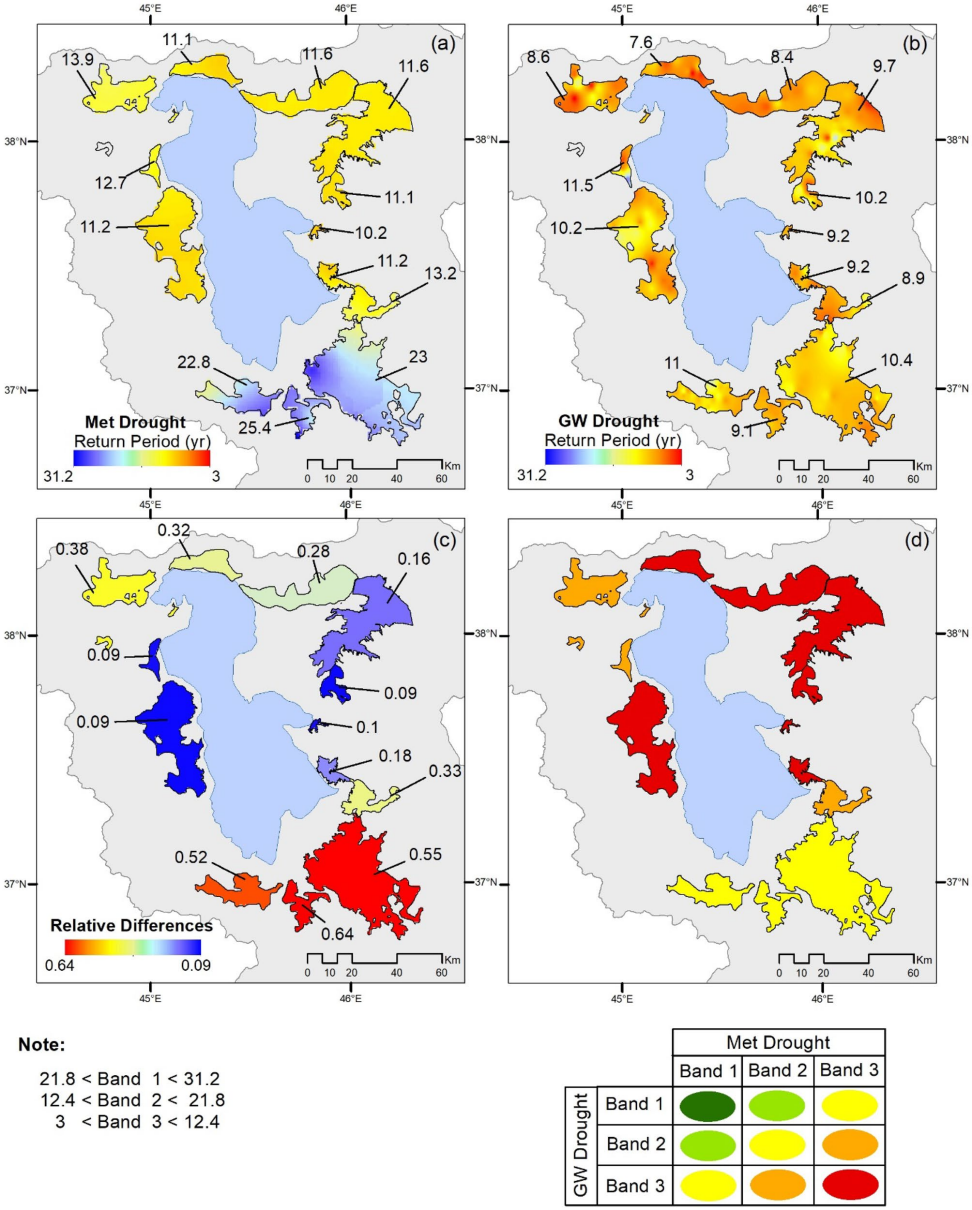
Variations in drought return periods between aquifers: (**a**) meteorological drought; (**b**) groundwater drought; (**c**) relative differences; (**d**) risk matrix derived for each aquifer based on return periods of Met/GW droughts. Note: The figure is produced by the authors using QGIS 3.01 v 2018.

The return period values produced by implementing the modelling strategy for Met/GW return periods are spatially distributed by the ordinary Kriging technique, the implementation of which is justified in the method section. The results are displayed in Fig. 4, which underpins the following broad findings:

(i) The return periods for the Met drought at the Southwest of the Basin are relatively high (and therefore less adverse regimes) in the region. This corresponds to the source of three dominant rivers with the potential to supply of more than 50% of the input surface flows to the lake in the old hydrological regime before its encroachment. Thus, at least, water levels at Lake Urmia should potentially be robust to adverse effects, if the flow regimes of these rivers are not encroached. However, the values of the Met return periods gradually decrease towards the southeast and northern parts of the basin (and therefore relatively more adverse drought regimes).
(ii) The GW return periods do not show significant differences from one aquifer to another, and this is apparent from the return period contours within the aquifers, where both droughts return periods use an identical colour pallet. Nevertheless, the comparison demonstrates a salient feature on return periods that for GW droughts, their values are detectably lower (more adverse) than those for Met droughts, and this signifies an intensified anthropogenic impact on drought occurrences.
(iii) The differences between the Met and GW droughts are wide among the aquifers and these are further shown in Fig. 5 as they have significant implications to be discussed next. Naturally, the rivers and aquifers in a basin exchange water through a gain/ loss process or mode, in which a river is recharged by its aquifers and hence the river is in its gain mode (but the aquifer is in the loss mode); whereas the aquifer is recharged by its river and hence the aquifer is in its gain mode (but the river is in the loss mode).

The authors’ observations on the above interconnectivities show that the rivers crossing the aquifers surrounding Lake Urmia used to display both modes in the course of past years. However, since the onset of anthropogenic activities and their intensification, the gain mode of the rivers has turned into a loss mode, and consequently the baseflows would hardly match their past states. There are a lack of comprehensive studies on the relationship between rivers and aquifers in the Lake Urmia basin, but this issue has been emphasized sporadically in authors’ previous studies^35^.

Informations obtained from soil texture and geological logs related to aquifers around the lake provide evidence that there are not significant interconnectivity between the lake and aquifers. So that the soil texture near the lake becomes clay and impermeable, although some limited interconnectivities have been noted for some of the aquifers, e.g. the Tasuj aquifer ^36^. The groundwater over-abstraction decrease surface inflow to the lake by changing rivers behaviour from the mode of gaining in an aquifer to the mode of losing to the aquifer. The paper indicates that this phenomenon becomes more probable due to the significant relative difference between Met and GW drought in some aquifers such as the Qoshachay (Miandoab) aquifer. The implication of this local knowledge is that the decline of Lake Urmia is not related to the aquifers but stems from the 40 embanking dams. Likewise, the depletion of the aquifers stems from over-abstraction by pumping.

The focus in Fig. 5 is on the aquifers, which uses an identical colour palette and ranges for the return period distributions of Met/GW droughts, where the range of the palette is from 3 to 31 years. It displays the mean values within the aquifers in the digital form on the top of solid lines. A comparison of Fig. 5a (Met droughts) with Fig. 5b (GW droughts) provides a visual evidence that GW droughts tend to be more frequent (lower values of return periods and more adverse) than those of Met droughts (higher return periods). These two visual observations are also supported statistically by a poor correlation coefficient between the mean return periods for Met/GW droughts, as follows. The value of the coefficient is 0.26 but this is not significant enough to underpin any significant dependence of return periods of GW droughts on those of Met droughts. The range of the return period of Met droughts is from 11 to 31 years but that of GW droughts is from 3 to 16 years and their relative differences range from 0.1 to 0.64 (the full range is from 0 to 1).

### Detecting anthropogenic impacts

GW droughts in the aquifers, displayed in Fig. 5, are the basis to extract the salient features on anthropogenic impacts by a combination of three techniques of visual inspection of the results in Fig. 5, studying the patterns in terms of the differences between return periods of Met and GW drought and working out a risk matrix for the aquifer. The results suggest the breaking down the aquifers into the following zones:

(i) *Return periods of Met droughts coincide with those of GW droughts*: This is not observed in this study area but it is argued that their coincidence should generally prevail in sustainable aquifers recharged annually by adequate amounts without triggering drought events.
(ii) *Northern Aquifers (Tasuj, Shabestar, and Salmas)*: The contours of both Met and GW droughts in this zone are within relatively severe (low values of return periods, see Fig. 5a, b) but their relative differences seem to be in the moderate range. The heuristic learning is that relative differences can be quite misleading as both return periods are relatively severe (low values of return period) but their differences diffuse their severity. Drought occurrences in these aquifers are further supported by the authors’ investigations on subsidence, who report that the subsequent subsidence is significant due to GW over-abstractions and may be considerable (e.g.,^12–14^).
(iii) *Aquifers at the Central Zones (eastern aquifers: Tabriz, Azershahr, Shiramin and Ajabshir; and western aquifers: Urmia and Kahriz):*The contours of both Met and GW droughts in this zone are relatively severe in terms of return periods (low values of return period), but those of GW droughts are only slightly severer than Met droughts. In these aquifers, anthropogenic activities are known to be intensive and therefore it is anticipated that as soon as they reach a biting point, the likely critical state of GW droughts could inflict a greater impact, if there is a failure on the ‘preparedness’ for droughts and the catastrophe of depleted aquifers. Relative values of mid-range droughts in Fig. 5c are justifiable, as high subsidence occurrences are yet to be observed in this zone.
(iv) *Aquifers of Southern Zones (Qoshachay-Miandoab, Mahabad, Sulduz-Naghade)*: The contours of Met droughts in this zone are within relatively low contours in terms of return periods (high values of return periods and less adverse), but those of GW droughts are in severe contours (low values of return periods and more adverse). These aquifers are already known to be stressed from severe anthropogenic impacts and Fig. 5c captures this salient feature.

A deeper understanding emerges by assessing a risk matrix for the area, which complements the above zones but with a different emphasis. The risk matrix (see,^10^) is a well-established risk management tool and its simplified application is captured in Fig. 5d, by breaking both Met and GW droughts into three bands of Band 1, Band 2 and Band 3. The likelihood of drought in each aquifer zone is qualified as *High* (Band3-Band3), *Fairly High* (Band3-Band2 or Band2-Band3), *Intermediate* (Band1-Band3, Band2-Band2 or Band3-Band1), *Fairly Low* (Band1-Band2 or Band2-Band1) and *Low* (Band1-Band1). Notably, the first occurrence of the band number refers to the return periods of Met Droughts and the second one to that of GW droughts. These results evidently corroborate with the observation and highlight the potential hotspots for droughts. There are some differences between the Fig. 5c and d, in which case for real project works, it is necessary to resort to the principle of precaution and select the worst cases.

The above results provide a heuristic basis to learn from the data on both visual grounds, and the analysis of differences and statistical coefficients that GW droughts are being impacted by anthropogenic encroachments more directly than meteorological processes. Arguably, this is a significant evidence for discerning anthropogenic impacts, as the local knowledge is of all aquifers are fast being depleted. However, the heuristic learning is that the resolution of dividing each return period to a number of bands need to be investigated. An adequate resolution should then be the base for devising an analytics for quantifying anthropogenic impacts. This may additionally require further dimensions and parameters into the inter-comparison, e.g. the capacity of an aquifer departing from its long-term recharged state).

The study employs an identical procedure to define return period for Met and GW droughts using monthly data but 3-month or 6-month SPI can normally be used too. However, the study seeks to cope with data sparsity with respect to the 21-year available data and therefore higher intervals would restrict the validity of frequency analysis. The above visual extraction of information using 21 years of data is fit-for-purpose but for the defensibility of the results need to be improved as more data becomes available.

## Methods

### Statistical distributions

The gamma distribution is used in the study by implementing its standard procedure with the two parameters, which comprises a shape parameter, *α*, and a scale parameter *β*, where both are positive real numbers (for more details, see^37^). Its parameter estimation procedure is inbuilt in the MATLAB platform and further details are available in the supplementary information file.

The exponential distribution is also used in the study by implementing its standard procedure with the one parameter, which comprises the rate parameter (*λ*), where it is a positive and real number (for more details, see^37^). Its parameter estimation procedure is inbuilt in the MATLAB platform and further details are available in the supplementary information file.

### Meteorological drought events

Further to the categorisation of drought studies in the introduction, the focus is now on Standardised Precipitation Index (SPI), developed by McKee et al.^5^, which is detailed next. It quantifies drought events based on precipitation timeseries for specific time scales, e.g., 1, 3, 6, or 12 months. To calculate SPI, a probability distribution is fitted to long-term precipitation data, and this renders the calculation of cumulative probabilities. More than often, the distribution fitted is the gamma distribution (see^5,38^). As per Eq. 1(a), the SPI values are defined in terms of the transformed inverse standard normal distribution (further details are available in ^39^):

1a $$SPI = \emptyset^{ - 1} \left( \alpha{\left( P \right)} \right)$$

where $\emptyset^{ - 1} \left( \cdot \right)$ represents the inverse standard normal distribution; and $\alpha\left( \cdot \right)$ is the gamma distribution fitted to precipitation timeseries (*P*). Notably, SPI varies in accordance with the x-axis range of standard normal distribution (approximately between − 3 to 3), and the negative values identify dry month. As per McKee et al. (1993) ^5^, the duration of a drought event is defined as a period in which SPI is continuously negative but its severity is defined as the cumulative magnitude of SPI for the duration (see Fig. 1).

### Groundwater drought events

Similarly, the focus is on index-based drought studies and the paper uses Standardised Groundwater level Index (SGI), developed by Bloomfield and Marchant^6^, which is processed by using changes in groundwater levels. The calculation procedure is similar to SPI (see Eq. 1b), but previous studies highlight that the monthly timeseries distribution for groundwater levels may not follow the gamma distribution and hence more distributions need to be evaluated ^6^. Therefore, the Gamma distribution was evaluated using the Kolmogorov–Smirnov test and the result confirmed the goodness-of-fit at 5% level of significance.

1b $$SGI = \emptyset^{ - 1} \left( {\beta \left( {GWL} \right)} \right)$$

where $\emptyset^{ - 1} \left( \cdot \right)$ represents the inverse standard normal distribution; and $\beta \left( \cdot \right)$ is an appropriate distribution fitted to groundwater level timeseries (*GWL*). Similar to SPI, the negative values for SGI identify the dry months. The duration and severity are also defined similar to SPI (Fig. 1).

### Copula

Copulas, are multivariate cumulative distributions normally applied for estimating the joint probability of multiple random variables, first developed in statistics by Sklar^40^ and then applied in hydrological studies such as rainfall-runoff^41^ and drought^42^. In drought studies, these functions calculate the probability of joint occurrences of drought characteristics such as duration and severity using marginal distribution functions. Marginal distribution functions indicate the probability of independent occurrence of drought characteristics ^34,43^. Shiau (2006) used Copulas to analyse the frequency of Met droughts, based on duration and severity characteristics using SPI^42^. The technique became topical with extensive reports (e.g.,^32,44^).

Recent applications of copulas include: (i) groundwater drought frequency analysis based on SGI^3^; (ii) conducting tri-variate (duration, severity, and peak)^45^ and fourth-variate (duration, severity, peak, and inter-arrival time)^46^ frequency analysis for Met droughts; (iii) uncertainty analysis in estimating copulas’ parameters using the Monte Carlo Markov chain^47,48^; (iv) combining different drought indices such as SPI with other drought indices such as the agricultural drought index^49^, the evaporative drought index^50^; and (v) drought frequency analysis based on the results of climate models ^51–54^.

A Copula function is a technique that indicates the relationship between a multivariate distribution function and one-dimensional marginal distributions. Copula models are based on the Sklar theory^40^, which use joint distributions for the random variable of *X* defined for *n*-dimensional continuous random variables (*X*_*1*_, *X*_*2*_, …, *X*_*n*_) with marginal distributions of F(*X*_*i*_) = P_x_(*X*_*I*_ < *x*_*i*_), as follows:

2 $$H_{{X_{1} , \ldots ,X_{n} }} \left( {x_{1} , \ldots ,x_{n} } \right) = {\text{P}}\left[ {X_{1} < x_{1} ,X_{2} < x_{2} , \ldots ,X_{n} < x_{n} } \right]$$

The bivariate copula (*C*) is defined as follows:

3 $$F_{XY} \left( {x,y} \right) = C\left( {F_{X} \left( x \right),F_{Y} \left( y \right)} \right)$$

where *x* and *y* are dependent random variables; *F*_*XY*_ is bivariate distribution function; and *F*_*X*_ and *F*_*Y*_ are marginal distributions.

The bivariate copula for drought duration and severity is defined as follows:

4 $$C\left( {u,v} \right) = F_{DS} \left( {d,s} \right) = C\left( {F_{D} \left( d \right),F_{S} \left( s \right)} \right)$$

where *d* and *s* are drought duration and severity, respectively; and *F*_*D*_ and *F*_*S*_ are marginal distributions for drought duration and severity, respectively (further information is available in^55^). In Met drought studies, previous research works indicate that the exponential distributions are appropriate for duration and the gamma distributions for severity^32,33,42^. In GW drought studies, there are no recommendations for the types of duration and severity probability distributions. Therefore, Gamma distribution was accepted using the Kolmogorov–Smirnov test at the 5% level of significance.

Table 3 illustrates the incorporated copulas in the study, which involves the estimation of copula parameters and for this, there are various techniques, including parametric, semi-parametric and non-parametric approaches. Among them, the Inference Function for Margins (IFM) method is the most common technique for estimating the copula parameter^56^ and requires two distinct steps outlined as follows. Step 1: marginal distributions are obtained from observed values; Step 2: the joint likelihood functions are maximised to estimate the copula parameter, *θ*. The logarithmic likelihood function is defined as follows:

5 $$L\left( {\theta} \right) = \mathop \sum \limits_{k = 1}^{n} {\text{log}}\left[ {c\left( {F_{D} \left( d \right),F_{S} \left( s \right)} \right)} \right] = \mathop \sum \limits_{k = 1}^{n} {\text{log}}\left[ {c\left( {u,v} \right)} \right]$$

where *n* is number of data; and *c* is the density function of copula and is calculated as follows ^57^:

6 $$c\left( {u,v} \right) = \frac{{\partial^{2} C\left( {u,v} \right)}}{\partial u\partial v}$$

**Table 3:** The list of incorporated copulas and related formulas.

Copula Family	Copula CDF ${ }C\left( {u,v} \right)$	Interval $\theta$
Clayton	$C\left( {u,v} \right) = \left( {u^{ - \theta } + v^{ - \theta } - 1} \right)^{ - 1/\theta }$	${\uptheta } \ge 1$
Ali-Mikhail-Haq	$C\left( {u,v} \right) = \frac{uv}{{1 - \theta \left( {1 - u} \right)\left( {1 - v} \right)}}$	$- 1 \le {\uptheta } \le 1$
Farlie-Gumbel-Morgenstern	$C\left( {u,v} \right) = \left[ {1 + \theta \left( {1 - u} \right)\left( {1 - v} \right)} \right]$	$- 1 \le {\uptheta } \le 1$
Frank	$C\left( {u,v} \right) = - \frac{1}{\theta }ln\left[ {1 + \frac{{\left( {e^{ - \theta u} - 1} \right)\left( {\left( {e^{ - \theta v} - 1} \right)} \right)}}{{\left( {e^{ - \theta } - 1} \right)}}} \right]$	${\uptheta } \ne 0$
Galambos	$C\left( {u,v} \right) = { }exp\left\{ { - \left[ { - \left( {lnu} \right)^{ - \theta } + \left( { - lnv} \right)^{ - \theta } } \right]^{{ - \frac{1}{\theta }}} } \right\}$	${\uptheta } \ge 0$
Gumbel-Hougaard	$C\left( {u,v} \right) = exp\left\{ { - \left[ {\left( { - lnu} \right)^{\theta } + \left( { - lnv} \right)^{\theta } } \right]^{{\frac{1}{\theta }}} } \right\}$	${\uptheta } \ge 1$
Plackett	${\text{C}}\left( {{\text{u}},{\text{v}}} \right) = \frac{1}{2}\frac{1}{{{\uptheta } - 1}}\left\{ {1 + \left( {\theta + 1} \right)\left( {u + v} \right) - \left[ {\left( {1 + \left( {\theta - 1} \right)\left( {u + v} \right)} \right)^{2} - 4\theta \left( {\theta - 1} \right)uv} \right]^{\frac{1}{2}} } \right\}$	${\uptheta } \ge 0$

### Joint return period

Two cases are considered for the return period: (i) simultaneous exceedance of both duration and severity from a given threshold, denoted by *T*_*DS*_; and (ii) exceedance of either duration or severity from a given threshold, denoted by ${T}_{DS}^{^{\prime}}$, as defined below^42^:

7 $$T_{DS} = \frac{E\left( L \right)}{{P\left( {D \ge d,S \ge s} \right)}} = \frac{E\left( L \right)}{{1 - F_{D} \left( d \right) - F_{S} \left( s \right) + C\left( {F_{D} \left( d \right),F_{S} \left( s \right)} \right)}} \left( {D \ge d\, {\text{AND}}\, S \ge s} \right)$$

8 $$T_{DS}^{^{\prime}} = \frac{E\left( L \right)}{{1 - C\left( {F_{D} \left( d \right),F_{S} \left( s \right)} \right)}} \left( {D \ge d\, {\text{OR}} \,S \ge s} \right)$$

where *L* is the interval between the onset of a drought to the next drought; and *E*(*L*) is the average of all *L* values.

### Empirical copula

The empirical copulas are order-based joint cumulative probabilities ^34^ and a two-dimensional copula with *n* number of observations (*C*_*e*_) is defined as follows:

9 $$C_{e} \left( {\frac{i}{n},\frac{j}{n}} \right) = \frac{{\# \left\{ {\left( {d,s} \right)|d \le d_{\left( i \right)} {\text{ and }}s \le s_{\left( j \right)} } \right\}}}{n}$$

where *d*_*(i)*_ and *s*_*(j)*_ represent the order statistics of observed duration and severity, $0\le i,j\le n$; and # denotes the cardinality of the identified sets.

### Specification of further techniques

The production of results includes the following techniques: (i) Root Mean Square Error (RMSE), Nash–Sutcliffe Efficiency (NSE) are used to select the best copula in addition to calculating the maximum logarithm likelihood. A ‘perfectly’ fitted copula has RMSE closer to zero and NSE, closer to 1. (ii) the calculated return periods in synoptic stations and observation wells are spatially distributed using the ordinary Kriging technique. The study employed a QGIS 3.01 v 2018 to implement the kriging method based on the concept of semivariogram to account for the spatial configuration of the sample points by using both the first and second moments of the measured data, which had the internal ability to set its parameters. Although the aim of the paper is not to investigate the capability of interpolation techniques, the literature review highlights the advantages of the kriging techniques over the others as follows: a relatively very small error probability, a relatively optimal smoothed contour line, and a small influence of very irregularly distributed station^58^.

### The modelling strategy

The modelling strategy in the paper is the key to be able to discern the role of anthropogenic impacts on aquifers. Whilst the theoretical groundworks are already laid down for estimating return periods of Met droughts, the paper tests the application of the procedure for GW droughts. The Introduction section outlined the salient features of the narrative for the modelling strategy. The modelling strategy is implemented in 9 steps and as illustrated in Fig. 1 and its description at each step is given below.

The strategy is as follows: **Step 1** –identify the spatial boundaries of the basin and aquifers and prepare data within the boundaries; data include precipitation data of synoptic stations within the Lake Urmia basin and also groundwater levels obtained from observation wells within the aquifers around the lake; **Step 2**—pre-process data to select or eliminate stations according to data length, conduct an outlier test based on the interquartile range^30^ and fill the gap in the data by using the data from the nearest stations with high correlation coefficient values; **Step 3**—calculate SPI values at 48 synoptic stations using Eq. (1a) and SGI values at 158 observation wells using Eq. (1b); **Step 4**—calculate duration and severity timeseries for Met/GW droughts at the investigated synoptic stations and observation wells; **Step 5**—fit the exponential distribution for duration, and the gamma distribution for severity; **Step 6**—identify the maximum durations and severities for Met/GW droughts at the investigated synoptic stations and observation wells; **Step 7**—examine the fitness of different copulas listed in Table 3 and select the best fitted one for the investigated synoptic stations and observation wells by optimizing the copulas’ parameter; **Step 8**—conduct frequency analysis to calculate return periods of Met/GW droughts for the estimated maximum severity and duration at the investigated synoptic stations and observation wells; **Step 9**—identify the role of anthropogenic activities in intensifying drought by (i) the relative difference between Met/GW droughts and (ii) risk matrix; and **Step 10**—spatially-interpolate the calculated return periods for Met/GW droughts.

The modelling strategy provide the basis for a heuristic study in the sense that there was no basis to anticipate the outcome of the study beforehand but their comparison guided by a number of simple rules/ observations born out of professional experience provided a sufficient basis to gain an evidence-based insight into a complex situation.

## Supplementary Information

Supplementary Information.
