# Supplementary material for: Investigating meteorological/groundwater droughts by copula to study anthropogenic impacts
Source: Sci Rep. 2022 May 18;12:8285. doi: 10.1038/s41598-022-11768-7 (PMC9117685; doi:10.1038/s41598-022-11768-7)
Supplement: Supplementary file 1 — Supplementary Information. [file 41598_2022_11768_MOESM1_ESM.docx]

**Estimating Parameters of Marginal Distributions**

The probability density function of exponential distribution is expressed as follows:

$f_{D}\left( d \right)=e^{-d} d>0$ (1)

where *d* is the drought duration and *λ* is a parameter.

The probability density function of gamma distribution expressed as follows:

$f_{s}\left( s \right)=\frac{s^{-1}}{{}\left( \right)}e^{{-s}/{}} s>0$ (2)

where *s* is drought severity; *α* and *β* are shape and scale parameters, respectively and are estimated from the observed data; and Γ is the gamma function.

The parameters of exponential and gamma distributions were estimated by the maximum likelihood and moment generating function methods, respectively. The cumulative drought duration and severity distribution functions are obtained as follows:

$F_{D}\left( d \right)=1-e^{-d}$ (3)

$F_{s}\left( s \right)=\int_{0}^{s} \frac{s^{-1}}{{}\left( \right)}e^{{-s}/{}}ds$ (4)

**Estimating the Exponential Distribution Parameter by the Maximum Likelihood**

The likelihood function is defined is expressed as follows (Taboga, 2017):

$L\left( \right)=\prod_{i=1}^{n} f\left( x_{i}; \right)=\prod_{i=1}^{n} \left( e^{-x_{i}} \right)={}^{n}\prod_{i=1}^{n} \left( e^{-x_{i}} \right)={}^{n}e^{-\sum_{i=1}^{n} x_{i}}$ (5)

In the logarithm form, the likelihood function is defined as follows:

$lnL\left( \right) =n\ln-\sum_{i=1}^{n} x_{i}$ (6)

Eq. (6) is differentiated with respect to λ and equated to zero, as follows:

$\frac{\partial lnL\left( \right)}{\partial}=\frac{n}{}-\sum_{i=1}^{n} x_{i}=0 =\frac{n}{\sum_{i=1}^{n} x_{i}}=\frac{1}{}$ (7)

where, $\bar{x}$is the mean of sample data.

**Estimating the Gamma Distribution Parameters by the Moment Generating Function Method**

The Gamma distribution is defined as follows:

$f\left( x \right)=\left\{ \begin{aligned} \frac{x^{-1}}{{}\left( \right)}e^{{-x}/{}}, &x>0 \\ 0 , &\mathrm{Otherwise} \end{aligned} \right.$ (8)

The Moment Generating Function of Gamma distribution is given by (Rahman et al., 2014):

${}_{x}\left( t \right)=\int_{-\infty}^{+\infty} e^{tx}.f\left( x \right)dx=\int_{0}^{+\infty} e^{tx}\frac{x^{-1}}{{}\left( \right)}e^{{-x}/{}}dx=\cdots$ (9)

$=\frac{1}{{}\left( \right)}\int_{0}^{+\infty} e^{x\left[ t-\frac{1}{} \right]}x^{-1}dx=\frac{1}{{}\left( \right)}L\left\{ x^{-1} \right\}$

The resulting integral can be solved by the Laplace transform and we know that the definition of Laplace transform is as follows (Taboga, 2017):

$L\left\{ f \right\}=\int_{0}^{+\infty} e^{-st}f\left( t \right)dt\underset{\Rightarrow}{for Gamma dist.}\left\{ \begin{aligned} s=\frac{1}{}-t \\ f\left( x \right)=x^{-1} \end{aligned} \right.$ (10)

Also, we know that:

$L\left\{ t^{k} \right\}=\frac{\left( k+1 \right)}{s^{k+1}} L\left\{ x^{-1} \right\}=\frac{\left( \right)}{s}=\frac{\left( \right)}{\left( \frac{1}{}-t \right)}$ (11)

${}_{x}\left\{ t \right\}=\frac{1}{{}\left( \right)}.\frac{\left( \right)}{\left( \frac{1}{}-t \right)}=\frac{1}{{{}\left( \frac{1}{}-t \right)}}=\left( 1-t \right)^{-}$ (12)

${}_{x}^{'}\left\{ t \right\}=-\left( - \right)\left( 1-t \right)^{--1}=\left( 1-t \right)^{--1}$ (13)

${}_{x}^{''}\left\{ t \right\}={\left( --1 \right)\left( - \right)\left( 1-t \right)}^{--2}=\left[ {}^{2}{}^{2}+{}^{2} \right]\left[ 1-t \right]^{--2}$ (14)

The first-order moment of the population around the origin of the coordinate is:

${}_{x}^{'}\left( t=0 \right)==\frac{1}{n}\sum_{i=1}^{n} x_{i}=\bar{x}$ (15)

The second-order moment of the population around the origin of the coordinate is:

${}_{x}^{''}\left( t=0 \right)={}^{2}{}^{2}+{}^{2}=\frac{1}{n}\sum_{i=1}^{n} {x_{i}}^{2}=\bar{x^{2}}$ (16)

Gamma distribution parameters can be calculated by solving two Eqs. (15) and (16) based on sample data.

Notably, the abovementioned procedure can be implemented by MATLAB through commands of “expfit(data)” and “gamfit(data)”.

**References**

Taboga, M. Lectures on Probability Theory and Mathematical Statistics - 3rd Edition. Independent Publishing Platform. ISBN: 9781981369195 (2017)

Rahman, G., Mubeen, S., Rehman, A., Naz, M. On k-Gamma and k-Beta distributions and Moment generating functions. *Journal of Probability and Statistics*, 982013, 6 (2014).

**
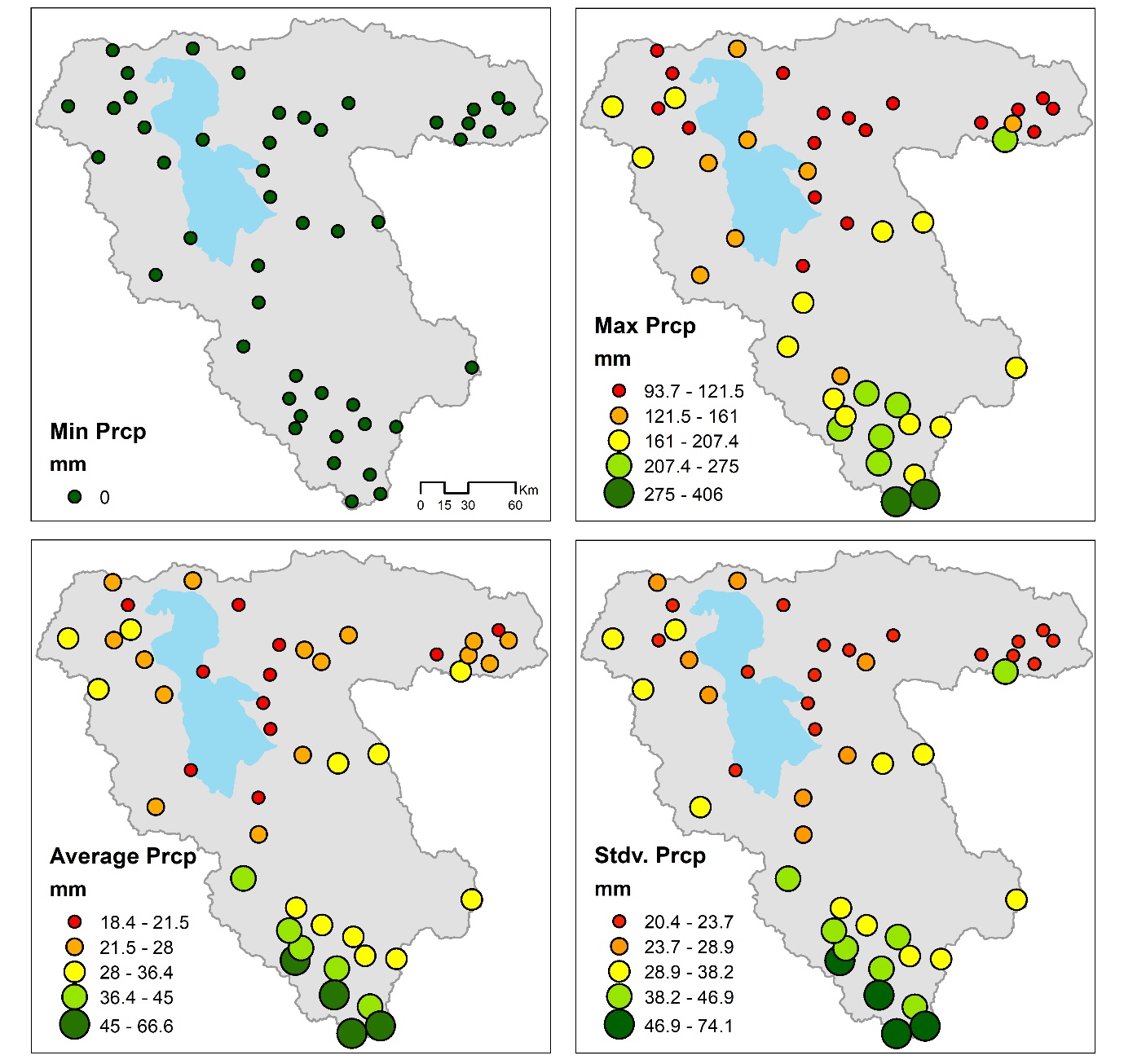
**

**Fig 1**. Statistical characteristics of synoptic stations

Note: The figure is produced by the authors using QGIS 3.01 v 2018.

**
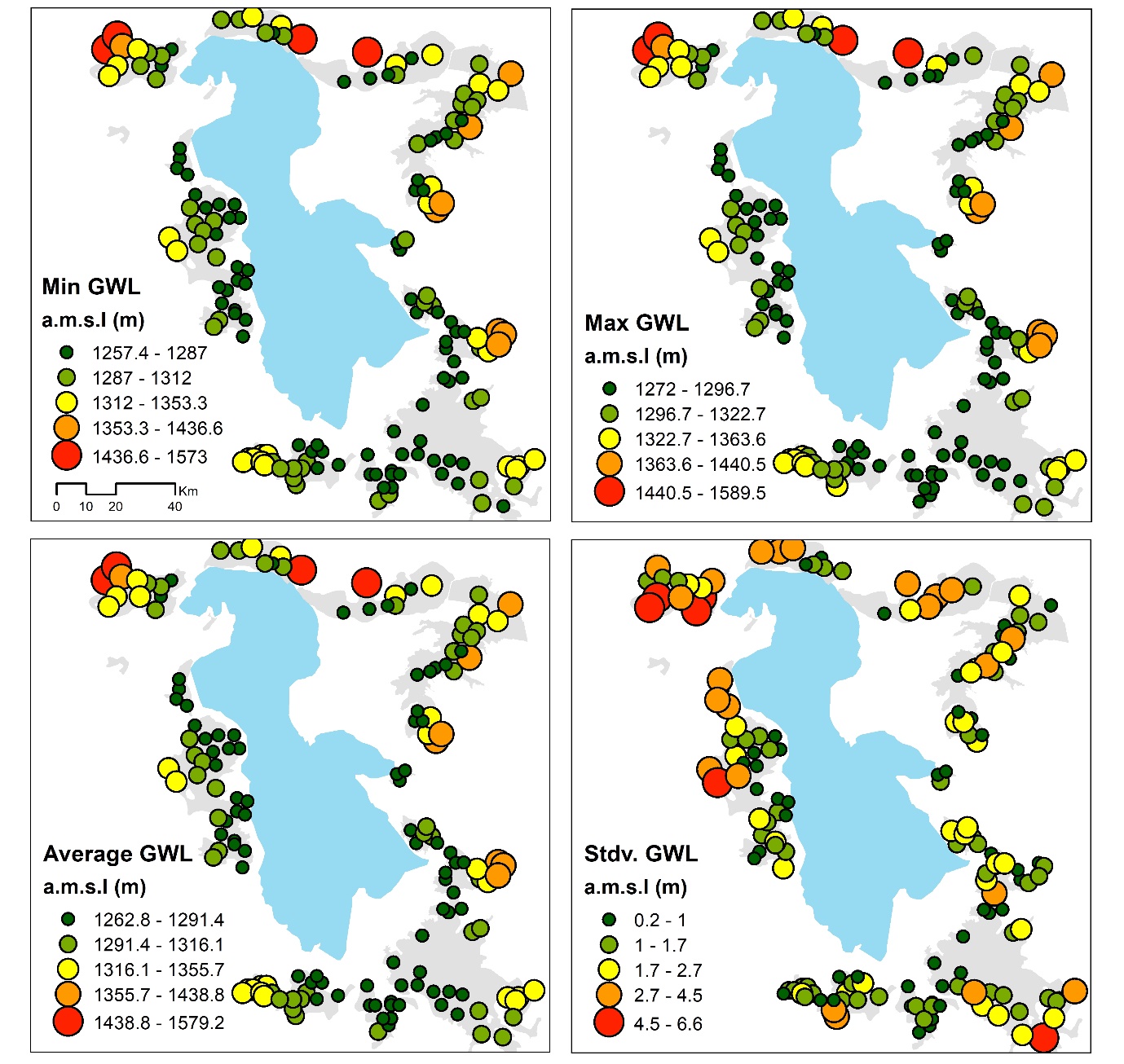
**

**Fig 2**. Statistical characteristics of observation wells

Note: The figure is produced by the authors using QGIS 3.01 v 2018.

**Table 1**. Statistical characteristics of synoptic stations

| Station  No. | Coordinate (degree) | | Min | Max | Statistical Period | Monthly Average | Standard Deviation | Skewness |
| --- | --- | --- | --- | --- | --- | --- | --- | --- |
|  | Long. | Lat. |  |  |  |  |  |  |
| 1 | 44.45 | 37.99 | 0 | 185.2 | 2000-2020 | 35.0 | 31.1 | 1.42 |
| 2 | 45.81 | 36.87 | 0 | 172 | 2000-2020 | 24.6 | 26.0 | 2.07 |
| 3 | 44.67 | 37.70 | 0 | 177.5 | 2000-2020 | 35.2 | 36.1 | 1.35 |
| 4 | 44.88 | 38.18 | 0 | 103.7 | 2000-2020 | 21.2 | 22.5 | 1.32 |
| 5 | 44.78 | 37.98 | 0 | 110.1 | 2000-2020 | 23.3 | 21.8 | 1.54 |
| 6 | 44.90 | 38.04 | 0 | 165.9 | 2000-2020 | 30.9 | 30.8 | 1.40 |
| 7 | 45.90 | 37.78 | 0 | 100.1 | 2000-2020 | 20.7 | 22.6 | 1.46 |
| 8 | 45.00 | 37.87 | 0 | 115.61 | 2000-2020 | 23.0 | 25.6 | 1.51 |
| 9 | 45.14 | 37.67 | 0 | 146.7 | 2000-2020 | 23.7 | 28.9 | 1.74 |
| 10 | 45.85 | 37.62 | 0 | 131 | 2000-2020 | 19.0 | 22.5 | 1.65 |
| 11 | 45.81 | 37.08 | 0 | 110 | 2000-2020 | 21.5 | 24.7 | 1.35 |
| 12 | 47.48 | 37.82 | 0 | 97.5 | 2000-2020 | 22.9 | 22.1 | 1.29 |
| 13 | 47.27 | 37.78 | 0 | 252.4 | 2000-2020 | 34.2 | 39.9 | 2.32 |
| 14 | 47.55 | 38.01 | 0 | 104.5 | 2000-2020 | 20.9 | 20.4 | 1.49 |
| 15 | 45.90 | 37.47 | 0 | 112.5 | 2000-2020 | 19.6 | 23.4 | 1.66 |
| 16 | 46.47 | 38.00 | 0 | 106.9 | 2000-2020 | 23.8 | 22.8 | 1.29 |
| 17 | 47.31 | 36.48 | 0 | 182 | 2000-2020 | 36.1 | 36.7 | 1.30 |
| 18 | 45.97 | 37.95 | 0 | 110 | 2000-2020 | 18.4 | 21.4 | 1.63 |
| 19 | 46.47 | 36.28 | 0 | 265 | 2000-2020 | 34.8 | 41.1 | 2.03 |
| 20 | 46.15 | 37.92 | 0 | 102.2 | 2000-2020 | 22.2 | 23.4 | 1.40 |
| 21 | 45.42 | 37.80 | 0 | 130.5 | 2000-2020 | 19.6 | 23.2 | 1.46 |
| 22 | 45.68 | 38.18 | 0 | 93.7 | 2000-2020 | 21.5 | 21.1 | 1.27 |
| 23 | 45.35 | 38.32 | 0 | 139.5 | 2000-2020 | 22.4 | 24.5 | 1.61 |
| 24 | 46.27 | 37.85 | 0 | 115 | 2000-2020 | 24.9 | 26.0 | 1.40 |
| 25 | 46.45 | 35.73 | 0 | 406 | 2000-2020 | 66.6 | 74.1 | 1.48 |
| 26 | 46.35 | 36.10 | 0 | 220 | 2000-2020 | 45.0 | 45.9 | 1.16 |
| 27 | 44.63 | 38.31 | 0 | 321 | 2000-2020 | 53.3 | 60.9 | 1.69 |
| 28 | 45.70 | 36.62 | 0 | 192.8 | 2000-2020 | 39.0 | 41.7 | 1.24 |
| 29 | 46.06 | 36.15 | 0 | 264.5 | 2000-2020 | 56.2 | 56.8 | 0.97 |
| 30 | 46.33 | 35.95 | 0 | 275 | 2000-2020 | 52.9 | 57.0 | 1.22 |
| 31 | 46.10 | 36.22 | 0 | 194 | 2000-2020 | 43.7 | 45.5 | 1.04 |
| 32 | 46.02 | 36.32 | 0 | 207.4 | 2000-2020 | 43.9 | 46.9 | 1.07 |
| 33 | 47.33 | 37.87 | 0 | 133 | 2000-2020 | 22.7 | 23.0 | 1.65 |
| 34 | 47.62 | 37.95 | 0 | 103 | 2000-2020 | 22.9 | 22.3 | 1.33 |
| 35 | 47.10 | 37.88 | 0 | 107 | 2000-2020 | 21.3 | 21.4 | 1.47 |
| 36 | 47.37 | 37.95 | 0 | 116 | 2000-2020 | 25.0 | 23.7 | 1.52 |
| 37 | 45.33 | 37.24 | 0 | 125 | 2000-2020 | 19.5 | 21.6 | 1.86 |
| 38 | 44.77 | 38.31 | 0 | 121.5 | 2000-2020 | 27.1 | 26.7 | 1.26 |
| 39 | 46.58 | 35.88 | 0 | 173.9 | 2000-2020 | 42.1 | 40.3 | 1.01 |
| 40 | 46.65 | 35.77 | 0 | 386.4 | 2000-2020 | 55.8 | 71.5 | 2.18 |
| 41 | 46.67 | 37.32 | 0 | 182.2 | 2000-2020 | 30.5 | 33.9 | 1.61 |
| 42 | 46.38 | 37.27 | 0 | 193.4 | 2000-2020 | 33.2 | 36.2 | 1.37 |
| 43 | 46.13 | 37.32 | 0 | 118.5 | 2000-2020 | 24.0 | 25.8 | 1.29 |
| 44 | 45.08 | 37.03 | 0 | 152 | 2000-2020 | 28.0 | 32.4 | 1.54 |
| 45 | 46.25 | 36.35 | 0 | 222.9 | 2000-2020 | 36.4 | 37.6 | 1.45 |
| 46 | 46.07 | 36.45 | 0 | 161 | 2000-2020 | 30.6 | 34.1 | 1.32 |
| 47 | 46.55 | 36.17 | 0 | 196.4 | 2000-2020 | 34.8 | 38.2 | 1.69 |
| 48 | 46.77 | 36.15 | 0 | 185.5 | 2000-2020 | 34.7 | 34.1 | 1.24 |

**Table 2**. Statistical characteristics of observation wells

| Aquifer | Coordinate (degree) | | Min | Max | Statistical Period | Average | Standard deviation | Skewness |
| --- | --- | --- | --- | --- | --- | --- | --- | --- |
|  | Long. | Lat. |  |  |  |  |  |  |
| Tasuj | 45.47 | 38.26 | 1501.9 | 1511.1 | 2000-2020 | 1504.6 | 1.5 | 1.28 |
|  | 45.23 | 38.32 | 1297.4 | 1308.8 | 2000-2020 | 1301.5 | 2.9 | 0.48 |
|  | 45.28 | 38.33 | 1338.7 | 1355.7 | 2000-2020 | 1344.0 | 3.3 | 0.95 |
|  | 45.39 | 38.30 | 1336.7 | 1342.6 | 2000-2020 | 1337.7 | 0.9 | 2.20 |
|  | 45.40 | 38.27 | 1292.2 | 1300.6 | 2000-2020 | 1295.0 | 1.6 | 0.58 |
|  | 45.36 | 38.28 | 1283.6 | 1291.0 | 2000-2020 | 1286.5 | 1.5 | 0.93 |
|  | 45.16 | 38.32 | 1294.7 | 1310.1 | 2000-2020 | 1300.4 | 3.0 | 0.49 |
|  | 45.33 | 38.28 | 1298.3 | 1300.1 | 2000-2020 | 1299.3 | 0.5 | 0.02 |
| Shabestar | 45.72 | 38.22 | 1573.0 | 1589.5 | 2000-2020 | 1579.2 | 3.1 | 0.98 |
|  | 45.83 | 38.18 | 1322.0 | 1337.0 | 2000-2020 | 1329.5 | 4.5 | 0.04 |
|  | 45.63 | 38.13 | 1282.8 | 1286.5 | 2000-2020 | 1284.6 | 0.9 | -0.22 |
|  | 45.83 | 38.15 | 1291.5 | 1295.2 | 2000-2020 | 1293.8 | 0.7 | 0.00 |
|  | 45.89 | 38.20 | 1277.0 | 1288.9 | 2000-2020 | 1279.7 | 3.0 | 1.74 |
|  | 45.80 | 38.15 | 1257.4 | 1274.0 | 2000-2020 | 1262.8 | 3.7 | 0.68 |
|  | 45.73 | 38.14 | 1261.3 | 1273.6 | 2000-2020 | 1265.2 | 2.6 | 0.95 |
|  | 45.97 | 38.21 | 1315.3 | 1321.3 | 2000-2020 | 1318.3 | 1.5 | 0.04 |
| Tabriz | 46.11 | 37.99 | 1381.7 | 1386.2 | 2000-2020 | 1385.3 | 0.5 | -3.38 |
|  | 46.27 | 38.15 | 1394.8 | 1396.2 | 2000-2020 | 1395.8 | 0.4 | -1.10 |
|  | 46.15 | 38.18 | 1305.8 | 1316.5 | 2000-2020 | 1313.2 | 1.8 | -0.49 |
|  | 46.05 | 37.95 | 1301.3 | 1310.0 | 2000-2020 | 1305.1 | 1.7 | 0.38 |
|  | 46.15 | 38.12 | 1322.5 | 1328.6 | 2000-2020 | 1325.5 | 1.4 | -0.50 |
|  | 46.08 | 38.06 | 1311.0 | 1314.9 | 2000-2020 | 1312.3 | 0.6 | 1.41 |
|  | 46.09 | 38.09 | 1312.0 | 1315.3 | 2000-2020 | 1313.3 | 0.7 | 0.55 |
|  | 46.22 | 38.10 | 1328.3 | 1333.6 | 2000-2020 | 1331.4 | 1.2 | -0.46 |
|  | 46.14 | 38.07 | 1311.4 | 1319.4 | 2000-2020 | 1316.1 | 1.4 | -0.43 |
|  | 45.98 | 37.96 | 1275.2 | 1283.0 | 2000-2020 | 1279.3 | 1.9 | -0.09 |
|  | 45.91 | 37.94 | 1290.3 | 1292.2 | 2000-2020 | 1291.4 | 0.4 | -0.15 |
|  | 46.05 | 38.01 | 1293.0 | 1304.2 | 2000-2020 | 1297.1 | 1.7 | 0.85 |
|  | 46.12 | 38.05 | 1291.2 | 1308.8 | 2000-2020 | 1301.2 | 4.0 | -0.09 |
|  | 46.02 | 37.97 | 1266.5 | 1283.4 | 2000-2020 | 1273.1 | 3.8 | 0.60 |
|  | 46.08 | 38.01 | 1282.8 | 1294.4 | 2000-2020 | 1288.8 | 2.4 | -0.05 |
|  | 45.96 | 37.95 | 1275.2 | 1283.0 | 2000-2020 | 1279.3 | 1.9 | -0.09 |
| Azershahr | 45.90 | 37.80 | 1270.1 | 1279.6 | 2000-2020 | 1275.0 | 1.9 | -0.28 |
|  | 45.98 | 37.74 | 1384.2 | 1391.6 | 2000-2020 | 1388.3 | 1.8 | -0.14 |
|  | 45.95 | 37.76 | 1333.7 | 1338.7 | 2000-2020 | 1335.7 | 1.1 | 0.41 |
|  | 45.96 | 37.81 | 1331.0 | 1332.5 | 2000-2020 | 1331.9 | 0.3 | -0.69 |
|  | 46.00 | 37.76 | 1436.6 | 1440.5 | 2000-2020 | 1438.8 | 0.9 | -0.40 |
|  | 45.93 | 37.80 | 1280.2 | 1288.0 | 2000-2020 | 1284.4 | 1.8 | 0.02 |
|  | 45.91 | 37.83 | 1279.2 | 1282.9 | 2000-2020 | 1280.9 | 0.6 | 0.10 |
| Shiramin | 45.84 | 37.62 | 1273.2 | 1280.7 | 2000-2020 | 1276.1 | 1.7 | 0.75 |
|  | 45.83 | 37.64 | 1273.0 | 1275.3 | 2000-2020 | 1274.5 | 0.4 | -0.07 |
|  | 45.86 | 37.65 | 1287.7 | 1291.1 | 2000-2020 | 1289.1 | 0.8 | 0.27 |

**Table 2**. Continued

| Aquifer | Coordinate (degree) | | Min | Max | Statistical Period | Average | Standard deviation | Skewness |
| --- | --- | --- | --- | --- | --- | --- | --- | --- |
|  | Long. | Lat. |  |  |  |  |  |  |
| Ajabshir | 45.96 | 37.45 | 1302.6 | 1309.5 | 2000-2020 | 1306.9 | 1.5 | -0.72 |
|  | 45.98 | 37.43 | 1275.0 | 1281.4 | 2000-2020 | 1278.3 | 1.3 | 0.50 |
|  | 45.90 | 37.43 | 1267.1 | 1273.7 | 2000-2020 | 1270.7 | 1.3 | -0.47 |
|  | 45.88 | 37.47 | 1271.1 | 1279.5 | 2000-2020 | 1274.7 | 1.9 | 0.54 |
|  | 45.93 | 37.46 | 1287.8 | 1299.1 | 2000-2020 | 1294.1 | 2.0 | -0.17 |
|  | 45.94 | 37.48 | 1303.2 | 1313.7 | 2000-2020 | 1310.2 | 2.4 | -1.06 |
| Maragheh-Bonab | 46.03 | 37.40 | 1275.1 | 1277.2 | 2000-2020 | 1276.1 | 0.4 | 0.09 |
|  | 46.21 | 37.37 | 1407.3 | 1411.3 | 2000-2020 | 1409.5 | 0.5 | 0.07 |
|  | 46.21 | 37.33 | 1353.3 | 1356.8 | 2000-2020 | 1355.1 | 0.8 | -0.03 |
|  | 46.04 | 37.28 | 1269.2 | 1280.7 | 2000-2020 | 1274.8 | 3.1 | 0.17 |
|  | 46.14 | 37.32 | 1306.7 | 1311.7 | 2000-2020 | 1310.1 | 0.9 | -1.19 |
|  | 46.23 | 37.36 | 1389.5 | 1396.0 | 2000-2020 | 1393.0 | 1.6 | -0.02 |
|  | 46.17 | 37.31 | 1322.7 | 1327.9 | 2000-2020 | 1325.4 | 1.2 | -0.14 |
|  | 46.01 | 37.32 | 1273.5 | 1283.2 | 2000-2020 | 1278.2 | 1.9 | 0.36 |
|  | 46.13 | 37.35 | 1318.1 | 1319.9 | 2000-2020 | 1319.0 | 0.3 | -0.07 |
|  | 46.21 | 37.33 | 1364.0 | 1370.0 | 2000-2020 | 1367.2 | 1.3 | -0.05 |
|  | 46.01 | 37.34 | 1275.8 | 1281.1 | 2000-2020 | 1278.3 | 1.2 | 0.06 |
|  | 46.05 | 37.37 | 1271.0 | 1280.3 | 2000-2020 | 1275.6 | 2.3 | -0.02 |
|  | 46.08 | 37.37 | 1281.6 | 1291.2 | 2000-2020 | 1285.0 | 2.5 | 0.73 |
| Qoshachay (Miandoab) | 45.92 | 37.15 | 1277.2 | 1280.5 | 2000-2020 | 1279.1 | 0.7 | -0.69 |
|  | 46.02 | 37.22 | 1277.8 | 1280.4 | 2000-2020 | 1278.6 | 0.6 | 0.69 |
|  | 46.00 | 37.23 | 1277.4 | 1278.5 | 2000-2020 | 1277.9 | 0.2 | 0.31 |
|  | 46.11 | 37.16 | 1294.7 | 1301.5 | 2000-2020 | 1298.0 | 1.3 | 0.14 |
|  | 46.14 | 37.17 | 1304.2 | 1312.5 | 2000-2020 | 1308.2 | 2.2 | 0.14 |
|  | 46.07 | 37.23 | 1276.4 | 1281.3 | 2000-2020 | 1278.7 | 1.0 | -0.07 |
|  | 46.28 | 36.96 | 1332.4 | 1336.4 | 2000-2020 | 1333.4 | 0.8 | 1.80 |
|  | 46.02 | 36.98 | 1280.4 | 1287.6 | 2000-2020 | 1284.4 | 1.5 | -0.35 |
|  | 45.81 | 37.07 | 1278.0 | 1280.7 | 2000-2020 | 1279.2 | 0.7 | 0.45 |
|  | 46.07 | 36.97 | 1285.0 | 1290.8 | 2000-2020 | 1288.2 | 1.4 | -0.21 |
|  | 45.90 | 37.00 | 1277.3 | 1283.1 | 2000-2020 | 1280.5 | 1.2 | -0.37 |
|  | 46.22 | 36.84 | 1285.6 | 1309.0 | 2000-2020 | 1300.2 | 6.1 | -0.74 |
|  | 46.14 | 36.85 | 1293.0 | 1300.8 | 2000-2020 | 1296.1 | 2.1 | 0.55 |
|  | 46.24 | 36.96 | 1315.3 | 1322.7 | 2000-2020 | 1319.2 | 1.6 | -0.38 |
|  | 45.99 | 36.94 | 1279.1 | 1289.1 | 2000-2020 | 1284.6 | 2.3 | -0.25 |
|  | 46.34 | 36.98 | 1349.9 | 1363.6 | 2000-2020 | 1354.6 | 3.3 | 0.40 |
|  | 46.13 | 36.92 | 1287.8 | 1294.8 | 2000-2020 | 1292.3 | 1.5 | -1.34 |
|  | 45.84 | 37.00 | 1277.8 | 1280.9 | 2000-2020 | 1279.5 | 0.7 | -0.07 |
|  | 45.96 | 36.98 | 1276.4 | 1286.9 | 2000-2020 | 1281.5 | 2.9 | 0.07 |
|  | 46.27 | 36.95 | 1323.3 | 1327.9 | 2000-2020 | 1325.0 | 1.2 | 0.43 |
|  | 45.91 | 37.04 | 1279.4 | 1283.9 | 2000-2020 | 1281.5 | 0.8 | 0.19 |
|  | 46.05 | 36.91 | 1278.9 | 1288.8 | 2000-2020 | 1285.0 | 2.2 | -0.62 |
|  | 46.26 | 36.90 | 1306.1 | 1312.8 | 2000-2020 | 1309.4 | 1.8 | -0.01 |

**Table 2**. Continued

| Aquifer | Coordinate (degree) | | Min | Max | Statistical Period | Average | Standard deviation | Skewness |
| --- | --- | --- | --- | --- | --- | --- | --- | --- |
|  | Long. | Lat. |  |  |  |  |  |  |
| Mahabad | 45.75 | 36.86 | 1290.7 | 1294.6 | 2000-2020 | 1293.5 | 0.9 | -0.75 |
|  | 45.79 | 36.88 | 1287.9 | 1291.8 | 2000-2020 | 1289.7 | 0.8 | -0.06 |
|  | 45.80 | 36.95 | 1278.0 | 1282.5 | 2000-2020 | 1280.5 | 1.0 | -0.05 |
|  | 45.80 | 36.90 | 1286.3 | 1290.5 | 2000-2020 | 1289.1 | 1.0 | -0.72 |
|  | 45.72 | 36.94 | 1281.4 | 1285.0 | 2000-2020 | 1283.6 | 0.8 | -0.44 |
|  | 45.71 | 37.00 | 1276.4 | 1278.4 | 2000-2020 | 1277.6 | 0.4 | -0.74 |
|  | 45.74 | 36.94 | 1277.7 | 1285.3 | 2000-2020 | 1283.2 | 1.4 | -0.68 |
|  | 45.80 | 36.94 | 1278.6 | 1284.1 | 2000-2020 | 1281.1 | 1.3 | 0.30 |
|  | 45.83 | 36.94 | 1278.0 | 1281.8 | 2000-2020 | 1280.1 | 0.7 | -1.02 |
|  | 45.77 | 36.90 | 1284.3 | 1289.8 | 2000-2020 | 1286.7 | 1.2 | 0.11 |
| Sulduz (Naghade) | 45.60 | 36.97 | 1279.6 | 1285.0 | 2000-2020 | 1282.9 | 1.1 | -0.66 |
|  | 45.48 | 36.98 | 1290.1 | 1296.7 | 2000-2020 | 1294.0 | 1.8 | -0.29 |
|  | 45.31 | 37.00 | 1320.5 | 1325.6 | 2000-2020 | 1323.9 | 1.2 | -0.79 |
|  | 45.50 | 37.01 | 1281.2 | 1288.2 | 2000-2020 | 1284.5 | 1.6 | 0.64 |
|  | 45.46 | 36.96 | 1294.6 | 1300.1 | 2000-2020 | 1296.9 | 1.2 | 0.59 |
|  | 45.45 | 37.03 | 1284.9 | 1287.8 | 2000-2020 | 1286.3 | 0.6 | 0.21 |
|  | 45.44 | 36.91 | 1309.1 | 1325.3 | 2000-2020 | 1315.1 | 3.7 | 0.45 |
|  | 45.33 | 37.00 | 1315.5 | 1321.9 | 2000-2020 | 1319.5 | 1.3 | -0.99 |
|  | 45.26 | 36.99 | 1333.5 | 1340.0 | 2000-2020 | 1338.2 | 1.2 | -1.49 |
|  | 45.43 | 36.93 | 1292.5 | 1303.8 | 2000-2020 | 1299.8 | 2.9 | -0.50 |
|  | 45.35 | 36.98 | 1307.9 | 1314.7 | 2000-2020 | 1312.2 | 1.2 | -1.03 |
|  | 45.43 | 36.96 | 1298.2 | 1302.6 | 2000-2020 | 1300.9 | 0.9 | -0.79 |
|  | 45.28 | 36.99 | 1325.1 | 1333.7 | 2000-2020 | 1331.8 | 1.4 | -2.15 |
|  | 45.24 | 36.98 | 1342.7 | 1347.6 | 2000-2020 | 1346.1 | 0.8 | -1.45 |
|  | 45.54 | 37.01 | 1281.0 | 1288.8 | 2000-2020 | 1285.0 | 2.1 | 0.18 |
|  | 45.31 | 36.98 | 1324.5 | 1331.8 | 2000-2020 | 1329.1 | 2.0 | -0.46 |
|  | 45.32 | 36.97 | 1320.4 | 1327.8 | 2000-2020 | 1325.0 | 1.7 | -0.65 |
|  | 45.52 | 36.96 | 1285.2 | 1291.0 | 2000-2020 | 1288.3 | 1.5 | 0.23 |
|  | 45.38 | 36.96 | 1301.9 | 1308.4 | 2000-2020 | 1305.9 | 1.0 | -1.50 |
|  | 45.52 | 37.03 | 1282.5 | 1286.3 | 2000-2020 | 1283.7 | 0.6 | 0.90 |
| Urmia | 45.06 | 37.70 | 1299.2 | 1309.9 | 2000-2020 | 1304.6 | 2.2 | 0.06 |
|  | 45.23 | 37.72 | 1275.7 | 1278.6 | 2000-2020 | 1277.3 | 0.7 | 0.07 |
|  | 44.96 | 37.66 | 1327.8 | 1349.2 | 2000-2020 | 1338.4 | 4.4 | 0.11 |
|  | 45.04 | 37.75 | 1295.9 | 1301.2 | 2000-2020 | 1297.7 | 1.2 | 0.72 |
|  | 45.13 | 37.71 | 1288.7 | 1293.4 | 2000-2020 | 1290.6 | 0.9 | 0.26 |
|  | 44.99 | 37.62 | 1328.9 | 1362.4 | 2000-2020 | 1343.6 | 6.3 | 0.02 |
|  | 45.15 | 37.41 | 1299.5 | 1303.1 | 2000-2020 | 1302.1 | 0.8 | -0.79 |
|  | 45.21 | 37.76 | 1273.8 | 1278.4 | 2000-2020 | 1276.4 | 0.9 | -0.29 |
|  | 45.09 | 37.68 | 1294.8 | 1298.4 | 2000-2020 | 1297.5 | 0.7 | -1.50 |
|  | 45.16 | 37.46 | 1284.7 | 1290.6 | 2000-2020 | 1289.1 | 1.5 | -0.92 |
|  | 45.15 | 37.76 | 1280.2 | 1287.1 | 2000-2020 | 1283.7 | 1.3 | -0.03 |
|  | 45.19 | 37.72 | 1277.2 | 1283.1 | 2000-2020 | 1280.6 | 1.5 | -0.31 |
|  | 45.07 | 37.64 | 1301.0 | 1315.9 | 2000-2020 | 1309.0 | 4.1 | -0.16 |

**Table 2**. Continued

| Aquifer | Coordinate (degree) | | Min | Max | Statistical Period | Average | Standard deviation | Skewness |
| --- | --- | --- | --- | --- | --- | --- | --- | --- |
|  | Long. | Lat. |  |  |  |  |  |  |
| Urmia | 45.10 | 37.75 | 1284.2 | 1293.0 | 2000-2020 | 1289.1 | 1.7 | -0.49 |
|  | 45.06 | 37.79 | 1273.5 | 1280.1 | 2000-2020 | 1276.0 | 1.8 | 0.60 |
|  | 45.13 | 37.39 | 1310.4 | 1314.6 | 2000-2020 | 1313.7 | 0.6 | -2.17 |
|  | 45.14 | 37.67 | 1284.0 | 1287.1 | 2000-2020 | 1285.7 | 0.8 | -0.28 |
|  | 45.22 | 37.53 | 1277.6 | 1284.0 | 2000-2020 | 1281.3 | 1.4 | -0.41 |
|  | 45.25 | 37.52 | 1274.8 | 1278.6 | 2000-2020 | 1276.8 | 0.9 | -0.06 |
|  | 45.14 | 37.60 | 1293.0 | 1295.1 | 2000-2020 | 1294.1 | 0.5 | -0.29 |
|  | 45.24 | 37.36 | 1270.6 | 1282.6 | 2000-2020 | 1277.6 | 2.7 | -0.33 |
|  | 45.18 | 37.50 | 1281.4 | 1289.2 | 2000-2020 | 1286.0 | 1.7 | -0.37 |
|  | 45.25 | 37.41 | 1274.1 | 1280.8 | 2000-2020 | 1278.0 | 1.5 | -0.23 |
|  | 45.22 | 37.57 | 1283.2 | 1286.3 | 2000-2020 | 1284.9 | 0.6 | 0.14 |
|  | 45.15 | 37.51 | 1287.0 | 1297.3 | 2000-2020 | 1292.6 | 2.6 | -0.21 |
|  | 45.21 | 37.44 | 1280.4 | 1288.0 | 2000-2020 | 1284.6 | 2.2 | -0.07 |
|  | 45.21 | 37.43 | 1282.8 | 1289.0 | 2000-2020 | 1286.8 | 1.5 | -0.21 |
|  | 45.26 | 37.56 | 1276.0 | 1278.9 | 2000-2020 | 1277.6 | 0.6 | -0.36 |
| Kahriz | 45.00 | 37.90 | 1268.3 | 1274.8 | 2000-2020 | 1270.8 | 1.3 | 0.61 |
|  | 45.00 | 37.93 | 1258.3 | 1272.0 | 2000-2020 | 1263.7 | 3.3 | 0.56 |
|  | 45.03 | 37.85 | 1263.3 | 1273.8 | 2000-2020 | 1268.0 | 3.0 | 0.28 |
|  | 44.99 | 37.87 | 1269.5 | 1287.2 | 2000-2020 | 1278.9 | 4.3 | -0.13 |
| Salmas | 44.93 | 38.18 | 1277.3 | 1294.9 | 2000-2020 | 1287.7 | 5.2 | -0.45 |
|  | 44.72 | 38.23 | 1482.2 | 1488.9 | 2000-2020 | 1484.7 | 1.4 | 0.27 |
|  | 44.76 | 38.27 | 1462.4 | 1476.2 | 2000-2020 | 1467.8 | 3.6 | 0.54 |
|  | 44.78 | 38.24 | 1383.6 | 1390.0 | 2000-2020 | 1386.1 | 1.4 | 0.44 |
|  | 44.97 | 38.23 | 1267.8 | 1284.7 | 2000-2020 | 1279.7 | 3.8 | -1.32 |
|  | 44.91 | 38.14 | 1288.7 | 1317.8 | 2000-2020 | 1302.3 | 6.6 | -0.49 |
|  | 44.76 | 38.18 | 1323.1 | 1346.2 | 2000-2020 | 1335.5 | 5.6 | 0.09 |
|  | 44.93 | 38.21 | 1290.6 | 1298.8 | 2000-2020 | 1294.7 | 2.5 | -0.04 |
|  | 44.88 | 38.22 | 1303.7 | 1311.5 | 2000-2020 | 1307.8 | 2.2 | -0.48 |
|  | 44.84 | 38.23 | 1325.7 | 1329.9 | 2000-2020 | 1327.6 | 1.1 | 0.02 |
|  | 44.85 | 38.18 | 1310.1 | 1326.7 | 2000-2020 | 1320.7 | 4.4 | -0.66 |
|  | 44.73 | 38.15 | 1343.4 | 1361.3 | 2000-2020 | 1355.7 | 5.2 | -1.22 |

**Table 3**. Kolmogorov-Smirnov test with 5% significance level (α) for some synoptic stations

| Station  No. | Coordinate (degree) | | Statistic | Critical value  α = 0.05 | Rejected or not? |
| --- | --- | --- | --- | --- | --- |
|  | Long. | Lat. |  |  |  |
| 1 | 44.45 | 37.99 | 0.045 | 0.091 | N |
| 3 | 44.67 | 37.70 | 0.044 | 0.096 | N |
| 4 | 44.88 | 38.18 | 0.053 | 0.099 | N |
| 7 | 45.90 | 37.78 | 0.065 | 0.099 | N |
| 8 | 45.00 | 37.87 | 0.065 | 0.099 | N |
| 11 | 45.81 | 37.08 | 0.051 | 0.103 | N |
| 14 | 47.55 | 38.01 | 0.054 | 0.094 | N |
| 15 | 45.90 | 37.47 | 0.061 | 0.102 | N |
| 17 | 47.31 | 36.48 | 0.093 | 0.093 | N |
| 20 | 46.15 | 37.92 | 0.094 | 0.096 | N |
| 21 | 45.42 | 37.80 | 0.072 | 0.102 | N |
| 23 | 45.35 | 38.32 | 0.066 | 0.093 | N |
| 24 | 46.27 | 37.85 | 0.043 | 0.097 | N |
| 26 | 46.35 | 36.10 | 0.081 | 0.100 | N |
| 27 | 44.63 | 38.31 | 0.041 | 0.096 | N |
| 33 | 47.33 | 37.87 | 0.060 | 0.094 | N |
| 34 | 47.62 | 37.95 | 0.051 | 0.094 | N |
| 35 | 47.10 | 37.88 | 0.040 | 0.096 | N |
| 36 | 47.37 | 37.95 | 0.063 | 0.093 | N |
| 37 | 45.33 | 37.24 | 0.046 | 0.098 | N |
| 39 | 46.58 | 35.88 | 0.071 | 0.099 | N |
| 40 | 46.65 | 35.77 | 0.058 | 0.101 | N |
| 41 | 46.67 | 37.32 | 0.048 | 0.100 | N |
| 42 | 46.38 | 37.27 | 0.094 | 0.097 | N |
| 46 | 46.07 | 36.45 | 0.094 | 0.096 | N |
| 47 | 46.55 | 36.17 | 0.058 | 0.099 | N |

**Table 4**. Kolmogorov-Smirnov test with 5% significance level (α) for some observation wells

| Aquifer | Coordinate (degree) | | Statistic | Critical value | Rejected or not? |
| --- | --- | --- | --- | --- | --- |
|  | Long. | Lat. |  |  |  |
| Tasuj | 45.28 | 38.33 | 0.082 | 0.093 | N |
|  | 45.40 | 38.27 | 0.048 | 0.094 | N |
|  | 45.16 | 38.32 | 0.053 | 0.096 | N |
|  | 45.33 | 38.28 | 0.055 | 0.094 | N |
| Shabestar | 45.83 | 38.18 | 0.092 | 0.093 | N |
|  | 45.83 | 38.15 | 0.038 | 0.093 | N |
|  | 45.80 | 38.15 | 0.088 | 0.095 | N |
|  | 45.73 | 38.14 | 0.096 | 0.101 | N |
|  | 45.97 | 38.21 | 0.073 | 0.096 | N |
| Tabriz | 46.15 | 38.18 | 0.068 | 0.093 | N |
|  | 46.05 | 37.95 | 0.055 | 0.093 | N |
|  | 46.22 | 38.10 | 0.071 | 0.092 | N |
|  | 46.14 | 38.07 | 0.055 | 0.093 | N |
|  | 45.98 | 37.96 | 0.049 | 0.092 | N |
|  | 45.91 | 37.94 | 0.057 | 0.093 | N |
|  | 46.05 | 38.01 | 0.065 | 0.094 | N |
|  | 46.12 | 38.05 | 0.059 | 0.094 | N |
|  | 46.08 | 38.01 | 0.063 | 0.094 | N |
|  | 45.96 | 37.95 | 0.061 | 0.096 | N |
| Azershahr | 45.90 | 37.80 | 0.046 | 0.093 | N |
|  | 45.98 | 37.74 | 0.058 | 0.092 | N |
|  | 45.96 | 37.81 | 0.089 | 0.092 | N |
|  | 45.93 | 37.80 | 0.075 | 0.096 | N |
|  | 45.91 | 37.83 | 0.074 | 0.096 | N |
| Shiramin | 45.83 | 37.64 | 0.051 | 0.094 | N |
|  | 45.86 | 37.65 | 0.082 | 0.096 | N |
| Ajabshir | 45.96 | 37.45 | 0.091 | 0.093 | N |
|  | 45.90 | 37.43 | 0.077 | 0.093 | N |
|  | 45.93 | 37.46 | 0.037 | 0.093 | N |
| Maragheh-Bonab | 46.21 | 37.37 | 0.086 | 0.093 | N |
|  | 46.21 | 37.33 | 0.060 | 0.093 | N |
|  | 46.14 | 37.32 | 0.073 | 0.093 | N |
|  | 46.01 | 37.32 | 0.044 | 0.093 | N |
|  | 46.13 | 37.35 | 0.055 | 0.093 | N |
|  | 46.21 | 37.33 | 0.041 | 0.098 | N |
|  | 46.01 | 37.34 | 0.041 | 0.098 | N |
|  | 46.05 | 37.37 | 0.041 | 0.095 | N |

**Table 4**. Continued

| Aquifer | Coordinate (degree) | | Statistics | Critical value | Rejected or not? |
| --- | --- | --- | --- | --- | --- |
|  | Long. | Lat. |  |  |  |
| Qoshachay (Miandoab) | 46.00 | 37.23 | 0.078 | 0.093 | N |
|  | 46.11 | 37.16 | 0.068 | 0.093 | N |
|  | 46.07 | 37.23 | 0.032 | 0.094 | N |
|  | 46.02 | 36.98 | 0.059 | 0.095 | N |
|  | 46.07 | 36.97 | 0.082 | 0.095 | N |
|  | 45.90 | 37.00 | 0.065 | 0.098 | N |
|  | 46.24 | 36.96 | 0.075 | 0.094 | N |
|  | 45.84 | 37.00 | 0.061 | 0.095 | N |
|  | 45.91 | 37.04 | 0.044 | 0.095 | N |
|  | 46.05 | 36.91 | 0.085 | 0.095 | N |
|  | 46.26 | 36.90 | 0.065 | 0.094 | N |
| Mahabad | 45.79 | 36.88 | 0.078 | 0.092 | N |
|  | 45.80 | 36.95 | 0.083 | 0.092 | N |
|  | 45.71 | 37.00 | 0.085 | 0.092 | N |
|  | 45.74 | 36.94 | 0.082 | 0.092 | N |
|  | 45.80 | 36.94 | 0.059 | 0.092 | N |
|  | 45.77 | 36.90 | 0.062 | 0.092 | N |
| Sulduz (Naghade) | 45.60 | 36.97 | 0.075 | 0.093 | N |
|  | 45.46 | 36.96 | 0.066 | 0.093 | N |
|  | 45.45 | 37.03 | 0.076 | 0.093 | N |
|  | 45.44 | 36.91 | 0.065 | 0.107 | N |
|  | 45.54 | 37.01 | 0.079 | 0.092 | N |
|  | 45.52 | 36.96 | 0.274 | 0.067 | N |
|  | 45.52 | 37.03 | 0.083 | 0.093 | N |
| Urmia | 45.06 | 37.70 | 0.045 | 0.094 | N |
|  | 45.23 | 37.72 | 0.053 | 0.094 | N |
|  | 45.13 | 37.71 | 0.052 | 0.094 | N |
|  | 44.99 | 37.62 | 0.047 | 0.094 | N |
|  | 45.21 | 37.76 | 0.061 | 0.094 | N |
|  | 45.15 | 37.76 | 0.053 | 0.094 | N |
|  | 45.07 | 37.64 | 0.071 | 0.095 | N |
|  | 45.25 | 37.52 | 0.041 | 0.097 | N |
|  | 45.14 | 37.60 | 0.061 | 0.097 | N |
|  | 45.24 | 37.36 | 0.063 | 0.097 | N |
|  | 45.25 | 37.41 | 0.068 | 0.097 | N |
|  | 45.22 | 37.57 | 0.068 | 0.097 | N |
|  | 45.26 | 37.56 | 0.048 | 0.097 | N |
| Kahriz | 45.00 | 37.90 | 0.062 | 0.102 | N |
|  | 45.00 | 37.93 | 0.080 | 0.094 | N |
|  | 44.99 | 37.87 | 0.056 | 0.094 | N |
| Salmas | 44.72 | 38.23 | 0.080 | 0.098 | N |
|  | 44.76 | 38.27 | 0.081 | 0.094 | N |
|  | 44.78 | 38.24 | 0.061 | 0.094 | N |
|  | 44.76 | 38.18 | 0.063 | 0.102 | N |
